# Non-Apoptotic Toxicity of *Pseudomonas aeruginosa* toward Murine Cells

**DOI:** 10.1371/journal.pone.0054245

**Published:** 2013-01-24

**Authors:** Sanhita Roy, Tracey Bonfield, Alan M. Tartakoff

**Affiliations:** 1 Department of Ophthalmology and Visual Sciences, Case Western Reserve University, Cleveland, Ohio, United States of America; 2 Department of Pediatrics, Case Western Reserve University, Cleveland, Ohio, United States of America; 3 Pathology Department and Cell Biology Program, Case Western Reserve University, Cleveland, Ohio, United States of America; The Scripps Research Institute and Sorrento Therapeutics, Inc., United States of America

## Abstract

Although *P. aeruginosa* is especially dangerous in cystic fibrosis (CF), there is no consensus as to how it kills representative cell types that are of key importance in the lung. This study concerns the acute toxicity of the sequenced strain, PAO1, toward a murine macrophage cell line (RAW 264.7). Toxicity requires brief contact with the target cell, but is then delayed for more than 12 h. None of the classical toxic effectors of this organism is required and cell death occurs without phagocytosis or acute perturbation of the actin cytoskeleton. Apoptosis is not required for toxicity toward either RAW 264.7 cells or for alveolar macrophages. Transcriptional profiling shows that encounter between PAO1 and RAW 264.7 cells elicits an early inflammatory response, followed by growth arrest. As an independent strategy to understand the mechanism of toxicity, we selected variant RAW 264.7 cells that resist PAO1. Upon exposure to *P. aeruginosa*, they are hyper-responsive with regard to classical inflammatory cytokine production and show transient downregulation of transcripts that are required for cell growth. They do not show obvious morphologic changes. Although they do not increase interferon transcripts, when exposed to PAO1 they dramatically upregulate a subset of the responses that are characteristic of exposure to g-interferon, including several guanylate-binding proteins. The present observations provide a novel foundation for learning how to equip cells with resistance to a complex challenge.

## Introduction

Although many microorganisms kill target cells by specific well-characterized mechanisms, for others the causes of toxicity are unknown. A case of particular interest is that of *P. aeruginosa*. This organism is acutely dangerous for individuals with cystic fibrosis (CF). Its large genome encodes multiple potentially toxic secretion systems [1], [2]. Moreover, it triggers multiple responses in host cells, e.g. [3], [4]. Adding to the complexity of the pathogen-host encounter are the changes of phenotype of the organism during infection, e.g. as a result of quorum sensing, differentiation into a mucoid state characterized by alginate production, establishment of biofilms, etc. [5]–[7].

Much attention has focused on the possible contribution to pathogenicity of protein secretion. Thus, the type III secretion apparatus injects bacterial enzymes into the target cell cytoplasm [8], [9] and the type II pathway releases multiple hydrolytic enzymes to the medium [10], [11]. Furthermore, *P. aeruginosa* lipopeptides and lipopolysaccharide (LPS) stimulate target cell Toll-like receptors, TLR2 and TLR4, respectively [12]. TLR4 signaling is proinflammatory, leading to nuclear translocation of NF-kB and activation of MAPK, while TLR2 signaling appears to oppose TLR4, e.g. [13]–[15]. Nevertheless, studies of knockout mice argue against a central role for TLR4 (or TLR2) in the acute pathogenesis that is characteristic of CF [16], [17].

Binding of *P. aeruginosa* to cell surfaces has been suggested to be mediated by the ganglioside GM1, fibronectin, integrins, and by the cystic fibrosis transmembrane regulator (CFTR). Internalization requires the kinases, PI3K and Akt, and the actin cytoskeleton [18], [19]. *P. aeruginosa* also produces two potentially toxic lectins [20].

To investigate the causes of toxicity by *P. aeruginosa*, we developed a highly sensitive assay using RAW 264.7 murine macrophage-monocytes as the target. Although human macrophages express surface CFTR, RAW 264.7 cells (and murine macrophages) do not [21], [22]. Reduced expression of CFTR is also characteristic of many cell type in cystic fibrosis [12], [23].

Among the issues of broader significance raised by the present observations is the question of what distinguishes inflammatory responses that are protective from those that damage target cells. We have generated variant cells that resist the toxicity of *P. aeruginosa*. Their transcriptional profiles, when compared to wildtype, show that many pro-inflammatory changes need not culminate in toxicity.

## Results

### 1. Characterization of Toxicity

#### A minimal toxicity assay for P. aeruginosa

**Figure 1:**
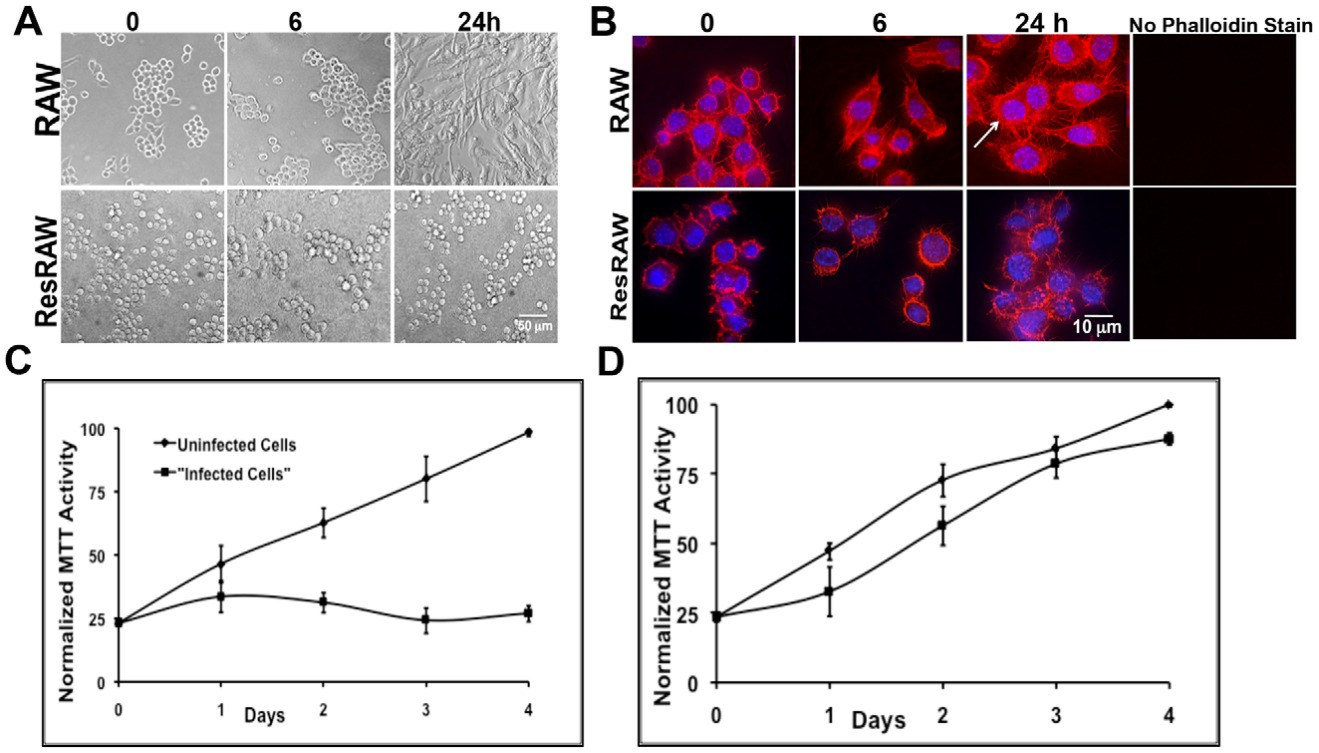
Impact of *P. aeruginosa* on viability on RAW 264.7 cells. **A**) Phase overview of cell morphology, uninfected cells (RAW) and resistant cells (ResRAW - see text) were exposed to *P. aeruginosa* (MOI 10) and returned to culture for 6 or 24 h. Note the major change in cell shape and size of the wt cells upon exposure to *P. aeruginosa*. The resistant cells appear unaffected over the same time period. **B**) Actin cytoskeleton, stained with rhodamine-phalloidin after fixation. Uninfected cells compared to resistant cells after exposure to *P. aeruginosa* (MOI 10) and reculture for 6 or 24 h. Note the enlargement of wt cells and appearance of a subset of cells with double nuclei (shown by an arrow). The nuclei were stained blue with DAPI. For the panels at the right, phalloidin staining was omitted and the images were viewed with the red filter. **C** and **D**: MTT assays to quantitate cell number. Uninfected cells compared to cells exposed to *P. aeruginosa* and then recultured over 4 days for both naïve RAW 264.7 (**C**) and resistant cells (**D**). In both cases, cells were exposed to *P. aeruginosa* for 1 h before washing and return to culture. Activity measurements (per culture) were normalized to values for cells cultured without PAO1 over four days.

Resting murine RAW 264.7 cells were exposed to the sequenced strain of *P. aeruginosa*, PAO1 - that is proficient in secretion of potentially toxic proteins [24] - for 1 h at low multiplicity of infection (MOI = 10). Cells were then washed and returned to culture in the presence of gentamicin and streptomycin, to eliminate extracellular *P. aeruginosa*. After 12 h, viability was still excellent, judging from MTT (3-[4,5-Dimethylthiazol-2-yl]-2,5-diphenyltetrazolium bromide) assays and the lack of release of cytoplasmic green fluorescent protein, GFP (when GFP-expressing target cells were used). Moreover, most cells had elongated and increased in size – as in many examples of macrophage activation (Figure 1A), and up to 25% of the cells had become binucleate [25] (Figure 1B). Nevertheless, the actin cytoskeleton still extended into filipodia (Figure 1B), distinguishing this response from that of cells that were exposed to higher multiplicities of *P. aeruginosa* or to other microorganisms, e.g. [9], [26], [27]. By 24 h, it was obvious that cell number and total MTT activity had not increased (Figure 1C). Moreover, in experiments with cells that expressed cytosolic GFP - ∼70% of the target cells were no longer fluorescent. Within 2–3 days the small number of remaining cells was severely vacuolated (not shown).

#### Contact is required for toxicity, but phagocytosis is not required

To learn whether contact between the bacteria and the host cell is required for toxicity, we ultracentrifuged bacterial cultures and passed the supernatant through a Millipore filter before diluting samples into media that were added to cell cultures for 1 h. Even at concentrations corresponding to MOI = 50, MTT assays and visual inspection showed no toxicity during the following days (not shown).

**Figure 2:**
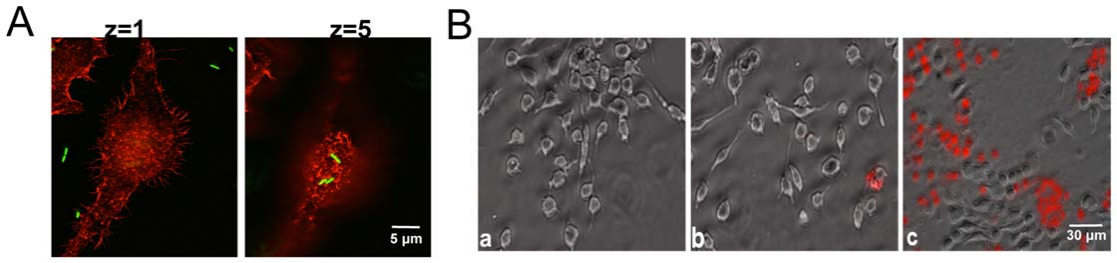
Morphological changes of naïve RAW 264.7 cells in response to PAO1. **A**) Fluorescent microscope images of RAW 264.7 cells exposed to PAO1 expressing GFP at MOI 10 for 1 h. After washing, cells were fixed and stained with phalloidin. Two different z planes are shown. **B**) Killing of RAW 264.7 cells exposed to PAO1 as indicated by appearance of propidium iodide stained cells after 48 h. a) Uninfected cells, b) Cells cocultured with cells exposed to PAO1, c) Cells directly exposed to PAO1.

By using PAO1 that expresses GFP in standard 1 h exposure toxicity protocols, we observed that only a small number of bacteria remained adherent to the filipodia after washing (Figure 2A). Upon reincubation for several hours, they then progressively deteriorated. We saw no visual evidence for internalization. The amount of internalization was also quantitated by washing the target cells after exposure to *P. aeruginosa*, preparing cell lysates under conditions that do not to kill the organism [28], plating the extracts on LB agar plates and incubating overnight. These experiments show that fewer than 1/20 of the target cells harbor a bacterium (not shown).

### 2. Requirements for toxicity

#### Toxicity is not due to factors released from RAW 264.7 cells exposed to PAO1

To learn whether soluble factors released from target cells cause toxicity, bacteria were incubated at MOI = 10 for 1–3 h with RAW 264.7 cells prior to collecting the medium, centrifuging it to remove bacteria, and Millipore filtration. The filtrates were then added to naïve cultures of RAW 264.7 cells for 1 h. Again, no toxicity was seen over the following days (not shown).

In parallel experiments, we performed cocultures in which a coverslip of cells was exposed to PAO1, washed, and then placed in a Petri dish that already contained a coverslip of naïve RAW 264.7 cells. After two days of culture with antibiotics, both coverslips were stained with propidium iodide: only the former showed evidence of toxicity (Figure 2B, panel c). The cells that never received bacteria but were cocultured with cells that had been exposed to bacteria (panel b) could not be distinguished from cells that were neither cocultured nor received any bacteria (panel a). Thus, contact between bacterium and target is required for toxicity and toxicity is cell-autonomous.

#### Type II and III Secretion

**Figure 3:**
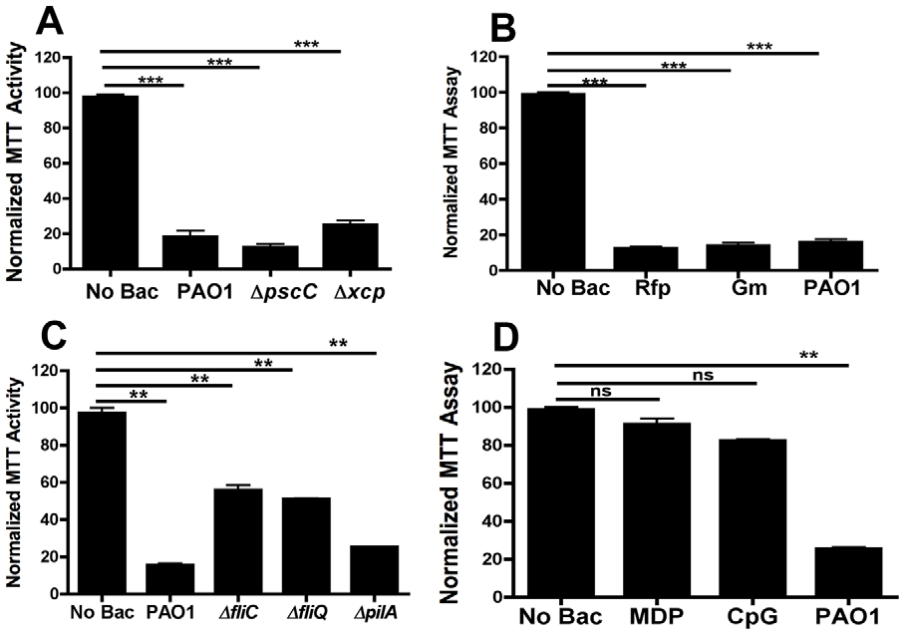
MTT assay to determine the viability of RAW 264.7 cells. RAW 264.7 cells were (**A**) exposed to Type II and Type III mutants of PAO1 for 1 h, or (**B**) to PAO1 in the presence of rifampicin (Rfp, 40 µg/ml), or gentamicin (Gm, 200 µg/ml). In (**C**), cells were exposed to different mutant strains of PAO1 containing mutations in pili and flagella. In (**D**), cells were exposed to muramyldipeptide (MDP, 25 µg/ml) or CpG DNA (5 µg/ml). In all cases cell viability was estimated by measuring MTT activity after 24–48 h as mentioned in text. (***) designates p<0.0003. ns: not-significant.

PAO1 produces several toxins. Some are secreted by its Type II secretion system, while the Type III system delivers others directly into the cytoplasm of target cells. To learn whether toxicity toward RAW 264.7 involves these systems, we tested the impact of two PAO1 mutants. The first lacks the type II secretion system (*xcp*), while the second lacks the type III secretion system (*pscC*) [29], [30]. Both mutants are as toxic as wildtype in our standard protocol (Figure 3A). A double mutant that lacks both systems is also obviously toxic (not shown).

#### Importance of bacterial metabolism

Toxicity appears to be due to components that are present at the time of addition of bacteria to the target cells. Neither inclusion of inhibitors of translation or transcription (Figure 3B) nor heat treatment (85°C, 60 min), has a major effect on toxicity. Taken together, we conclude that the bacteria do not need to be metabolically active to cause toxicity. We therefore investigated the importance of surface components of *P. aeruginosa*.

#### Surface components, lectins, muramyldipeptide, DNA

To learn whether appendages contribute to toxicity, we tested the impact of PAO1 mutants that lack flagella (*ΔfliC*, *ΔfliQ*) or pili (*ΔpilA*). The known pro-inflammatory impact of flagellin is mediated by TLR5, that binds bacterial flagellin [31], that is not expressed on RAW 264.7 [17], [32]. All three mutants were clearly toxic (Figure 3C).

Lectins of *P. aeruginosa* bind preferentially to galactose and fucose and could contribute to toxicity. We therefore conducted standard assays in the absence or presence of fucose (50 mM), galactose (50 mM), p-nitro-phenyl-fucoside (25 mM) and IPTG (0.5 mM), both singly and in combination with each other. No reduction in toxicity was detected (not shown).

Muramyldipeptide (MDP), a minimal structural unit of peptidoglycan, is present in the outer wall of Gram-positive bacteria and Gram-negative bacteria and is known to stimulate the immune system [33]. When RAW 264.7 cells were treated with high doses of MDP for 2 days there was no evidence of cell death (Figure 3D).

Proinflammatory stimulation by bacterial DNA is mediated by TLR9, that resides in endocytic compartments, e.g. [34]. The TLR9 ligand, CpG DNA, was therefore tested for toxicity; however, a high dose of B type CpG DNA caused only modest toxicity over 2 days (Figure 3D).

#### Lipopolysaccharide

The major *P. aeruginosa* surface-associated virulence factor, lipopolysaccharide (LPS), plays an important immunogenic and structural role, mediates interactions between the bacterial cell surface and the external environment, and binds TLR4 [35]. To evaluate the contribution of LPS to toxicity, we challenged cells with graded doses of soluble LPS purified from *P. aeruginosa*, serotype 10**.** Continuous exposure for 5 days is toxic; however, the LD_50_ for such preparations is at least 100× higher than the amount of LPS that we calculate is present on bacteria at MOI = 10 (Table 1). This could reflect the increased impact of clustering of binding sites on the target cell.

**Table 1:** Overview of Transcript Changes.

Condition	Increases		Decreases	
	Categories		Categories	
Wt cells+P.a. 1 h	apoptosis, inflammatory response, immune response, positive regulation of the I-kB kinase-NF-kB cascade	p<10^−11^	modest	marginal
Wt cells+P.a. 1+8 h	apoptosis, inflammatory response, immune response, positive regulation of the I-kB kinase-NF-kB cascade, positive regulation of cell proliferation, chemotaxis, anti-apoptosis, neutrophil chemotaxis, regulation of apoptosis, response to LPS, PDGF receptor signaling pathway	p<10^−10^	cell cycle progression, cell division, mitosis, cell cycle regulation, nucleosome assembly	p<10^−13^
mutant cells vs wt	signal transduction, prostaglandin biology	10^−6^<p<10^−3^	immune and inflammatory responses	10^−7^<p<10^−3^
Mutant+P.a. 1 h	apoptosis, immune response, inflammatory response, positive regulation of I-kB kinase-NF-kB cascade, anti-apoptosis	10^−16^<p<10^−6^	various	10^−16^<p<10^−6^
Mutant+P.a. 1+8 h	apoptosis, immune response, inflammatory response, positive regulation of I-kB kinase-NF-kB cascade, anti-apoptosis		cell cycle progression, cell division, mitosis, cell cycle regulation, nucleosome assembly	10^−22^<p<10^−14^
Wt: 1+8 h vs 1 h	Ccl2, Cd274, Edn1, Gbp1, Il1f6, Isg15, Rsad2, and Serpinb9		Little change	
Mutant: 1+8 h vs 1 h	Many g-interferon targets, Rsad2, Gbp1, Isg15, Gbp3, Gbp5, Gbp6, Ifi202b, tenascin		Little change	

The toxicity of LPS is generally thought to be due to its lipid A moiety [35]. Consistent with this information, we observe that *P. aeruginosa* expressing LPS with truncated glycans is also potent (Figures S1A and S1B).

As a further test of the contribution of LPS to toxicity, we evaluated the possible protective effect of polymyxin B, an agent that sequesters LPS in a stoichiometric complex [36]. We find that polymyxin B is an effective inhibitor of the toxicity of soluble LPS; however, it provides only minimal protection against PAO1 (Figure S1C).

### 3. Response to *P. aeruginosa*

#### NF-kB Activation

**Figure 4:**
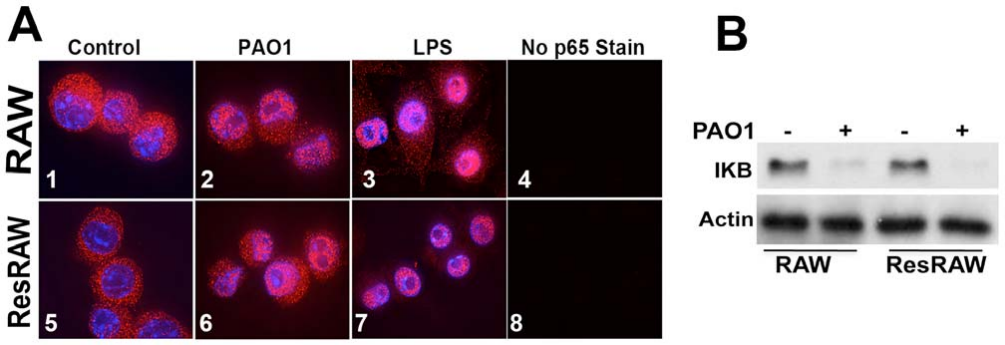
NF-kB activation in naïve and resistant RAW 264.7 cells. **A**) Naïve and resistant RAW 264.7 cells (ResRAW) were exposed to PAO1 (MOI 10) or to LPS (100 ng/ml) for 1 h. Note the translocation of the p65 subunit of NF-kB to the nucleus in both cell types. For the right-hand most images, preparations that received no primary antibody were viewed in the red channel. **B**) To quantitate the inhibitor of NF-kB (IKB), cells were exposed to PAO1 for 1 h, harvested and processed for Western blot analysis to detect IkB and b-actin. Note the major reduction in total IKB.

Nuclear factor-kB (NF-kB) is a critical participant in the immediate early pathogen response, regulating inflammation, cell proliferation and survival. In resting cells, NF-kB is bound to cytoplasmic inhibitors (IkBs), while it translocates to the nucleus in response to various stimuli, e.g. [37]. As expected, we observe that the p65 subunit of NF-kB enters the nucleus within one hour of exposure to bacteria (Figure 4A, 4B). It is excluded from the nucleolus and from the most chromatin-rich regions of the nucleoplasm, judging from preparations stained with DAPI. Treatment of cells with soluble LPS also causes nuclear translocation of p65 (Figure 4A). As shown in Figure 4B, total IkB decreases in RAW cells upon 1 h exposure to PAO1. Parallel assessment of phosphorylation of IkB shows a variable increase (not shown).

#### Cytokine Profiles

**Figure 5:**
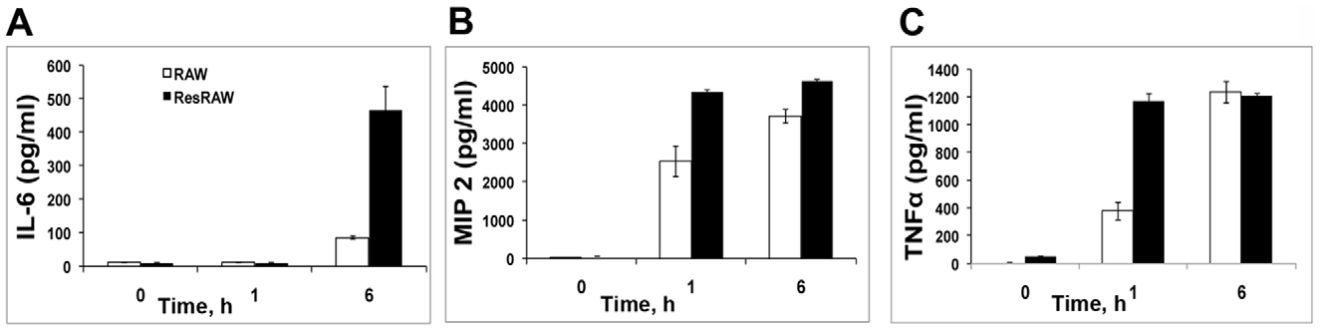
Cytokine production in response to PAO1. IL6 (**A**), MIP2 (**B**) and TNFα (**C**) secretion by naïve (solid line) and resistant (dotted line) RAW 264.7 cells were quantitated by ELISA, as described in Methods. Cells were exposed to PAO1 at MOI 10 for 1 h and recultured. Supernatants were collected at 0, 1 and 6 h. Cytokine production of mean +/− SD of three wells for each condition.

Supernatants harvested at 1, 6 and 24 h of exposure to PAO-1 were assayed for IL-6, MIP-2, and TNFα, secretion. Figure 5 shows that MIP-2 and TNFα were significantly elevated as early as 1 h, consistent with the rapid activation of NF-kB. IL-6 secretion became obvious by 6 h (Figure 5).

#### Transcriptional changes [Note: For references pertaining to specific proteins see Table S3.]

We recovered RNA for microarray analysis from cells before exposure to PAO1, after 1 h exposure, and after exposure for 1 h followed by washing and 8 h reculture with antibiotics (“1+8 h”). *P. aeruginosa* is known to elicit major transcriptional changes in other target cells, e.g. [38]–[40]. Table 1 (and Table S2) provide an overview of these data. Both Tables are based on the gene ontology analysis.

After 1 h exposure, changes by comparison to t 0 are summarized in Table S5A. In Table S6A the data have been trimmed to remove unannotated genes and changes of lesser statistical significance. Table S7(1d) and S7(1i) group the entries in Table S6A according to gene ontology/pathway criteria, where the suffixes (i) and (d) designate tabulations of the increases *vs* decreases.

Categories that show major increase after 1 h exposure (Table S7(1i)) include (p<10^−11^): apoptosis, inflammatory response, immune response, and positive regulation of the I-kB kinase-NF-kB cascade. Specific transcripts encode cytokines that we have measured (MIP-2 and TNFα), as well as many others (CCL2, CCL4, CCL5, CCL7, Csf3, CXCL3, CXCL10, Edn1, IL1a, IL1b, Il1f6 and Lif). Most of these increases are also seen at 1+8 h (Table S7(2), S6C/D). Those that appear only at +1 h are the transcription factor, Bhlhb2, Irf4 (that negatively regulates NF-kB signaling), and the TNF family member, Tnfsf9. Decreases seen only at +1 h are for two little-characterized g-interferon-responsive proteins, Ifi27 and Ifi44. None of the decreases have p-values that are nearly comparable to those of the increases.

Categories that increase after 1+8 h (p<10^−10^) (S7(2i)) include those noted after 1 h, with addition of: positive regulation of cell proliferation, chemotaxis, anti-apoptosis, neutrophil chemotaxis, regulation of apoptosis, response to LPS, and PDGF receptor signaling pathway.

The group that increases >10x includes selected cytokines (Ccl2, Il1f6) and, interestingly, some responses that can be triggered by g-interferon (CD274, endothelin-1, Gbp1, Isg15, Rsad2/viperin, Serpinb9). Since there is no increase in g-interferon transcripts, these latter changes are likely to reflect cross-talk between pathways.

Categories that decrease after 1+8 h show greater statistical significance (p<10^−13^) than increases (Tables S7(2i/d)). Thus, many decreases reflect strong inhibition of cell proliferation: cell cycle progression, cell division, mitosis, cell cycle regulation and nucleosome assembly. The group of strongest decreases encodes many histones, ribosomal proteins, subunits of the mitochondrial Fo-ATPase, and the actin-binding protein, anillin

Transcripts that are most strongly downregulated at 1+8 h by comparison to 1 h, as is evident in the categories, include many that are required for cell growth (Table S7(2)). Table S6C lists transcripts that are changed at 1+8 h but not more than 2x at 1 h.

Among entries that decrease selectively at 1 h compared to 1+8 h (Table S6D), none show changes of >10x.

### 4. Mechanism of Toxicity

#### What signaling pathway mediates toxicity?

**Figure 6:**
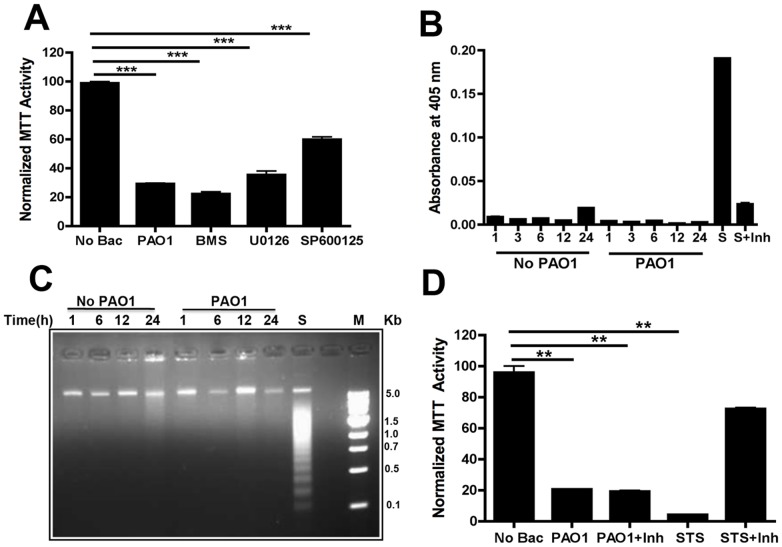
Toxicity of PAO1 toward RAW 264.7 cells. **A**) RAW 264.7 cells were exposed to PAO1 in the presence of different kinase inhibitors: SP600125 (for JNK, 10 mg/ml), U0126 (for MEK, 10 mg/ml) and BMS345541 (for IKK, 10 mg/ml). MTT assays quantitated cell viability after 1 day. **B**) RAW 264.7 cells were treated with PAO1 (MOI 10, 1 h) and washed. Caspase-3 activity was measured by colorimetric assays after 1–24 h. Control cells not exposed to bacteria were collected at the same time points. Sample treated with staurosporine (STS, 1 µg/ml) with or without caspase-3 inhibitor (Inh) for 3 h were used as a positive control. **C**) RAW 264.7 cells were exposed to PAO1 (MOI 10) for 1 h. DNA was isolated from cells at the indicated time points and analyzed by agarose gel electrophoresis. Control cells were not exposed to PAO1. Cells incubated with staurosporine (S) for 3 h were used as a positive control. M denotes the DNA ladder. **D**) RAW 264.7 cells were exposed to PAO1 +/− prior incubation with caspase 3 inhibitor (Inh). The viability of the cells was measured by MTT assay after 2 days. Cells treated with staurosporine were used as a positive control. Data are indicative of three repeat experiments with similar results and error bars represent standard deviations. (***) designates p<0.0003.

To examine the role of signaling pathways in cell death, we monitored the possible protective effect of adding kinase inhibitors 3 h before PAO1 treatment. SP600125, that inhibits the phosphorylation of c-Jun and expression of inflammatory genes, reduced cell death by almost 50% at 10 µg/ml (Figure 6A). Other inhibitors, U0126 (for MEK) and BMS345541 (for IKK), had little effect.

#### A Role for Apoptosis?

Many host-pathogen interactions are linked to activation of cascades that converge on caspase 3, stimulating both extrinsic and intrinsic pathways, e.g. [41], [42]. We therefore evaluated caspase 3 activity after exposure to PAO1. Negative controls (unstimulated cells) and a positive control consisting of cells treated with staurosporine, were analyzed in parallel. As expected, staurosporine strongly increased activity, but no increase was detected even after 24 h in cells exposed to PAO1 (Figure 6B). Consistent with this apparent lack of involvement of apoptosis, in protocols that show obvious DNA laddering after treatment with staurosporine we do not see laddering for cells treated with PAO1, even after 24 h (Figure 6C).

To learn whether toxicity nevertheless could be due to apoptosis, we added the cell-permeable caspase-3 inhibitor, Ac-DEVD-CHO, 4 h prior to addition of bacteria and also included it during the subsequent 1–2 day incubation. As illustrated in Figure 6D, this inhibitor blocked killing by staurosporine but had no effect on the toxicity of *P. aeruginosa*. Parallel experiments show that murine alveolar macrophages are also killed by *P. aeruginosa* and that that apoptosis is not involved (Figure S2). Possibly, although DNA cleavage and internalization of *Pseudomonas* do not appear to be involved, *Pseudomonas* toxicity is mechanistically related to necrosis or pyroptosis [43]–[46].

### 5. Selection of resistant cells

To further explore the mechanism of toxicity, we recovered RAW 264.7 mutants that resist *P. aeruginosa*. Thus, unmutagenized cells were exposed to wild type bacteria for 1 h at MOI = 10, washed, and then recultured for 4 days, at which point the majority of the cells had died. The survivors were allowed to recover, and were again exposed to *P. aeruginosa* for 1 h. After ten iterations of this protocol the survivors tolerated exposure to *P. aeruginosa* to a much greater extent than naïve cells (Figure 1D). Their resistance is thus a stable characteristic. Although we have not proven that these cells carry chromosomal mutations, we refer to them as “mutants.” They also are much more resistant than naïve cells when exposed to *P. aeruginosa* for as long as 5 h at MOI 50 (not shown).

We recovered five clones of resistant cells by limiting dilution. Since they all originated from a single pool of cells, they could be identical to each other. We do not think that they preexisted among the naïve cells, since exposure of naïve cells to doses of *P. aeruginosa* that are tolerated by the mutants totally eradicates the naïve population. In the following studies we focus on one clone (#2).

### 6. Characterization of Mutants

#### Morphology, cross-resistance

**Figure 7:**
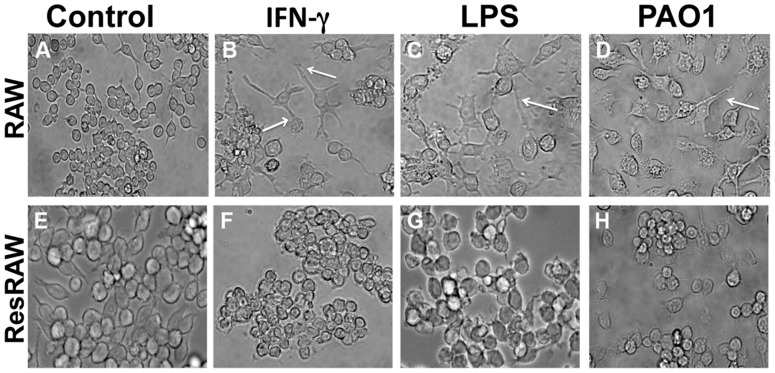
Morphological changes of naïve and resistant RAW 264.7 cells. Both naïve and resistant cells were exposed to g-interferon (25 ng/ml), soluble LPS (2 mg/ml) and PAO1 (MOI 10). After 1 h they were returned to culture for 48 h. The large differences of responsiveness of between the naïve and resistant cells are indicated by arrows. The naïve cells upon exposure to g-interferon, LPS, and PAO1 have extended processes and appear vacuolated, whereas the appearance of the resistant cells upon treatment does not differ from that of untreated control cells.

Upon treatment with PAO1, soluble LPS of *P. aeruginosa*, or mouse g-interferon for 24 h, wild type cells spread and (especially with g-interferon), extend processes (Figure 7B), and ultimately become highly vacuolated (Figure 7C and 7D). By contrast, the mutant cells do not change shape or size or generate binucleated cells upon exposure to *P. aeruginosa* (Figure 1A). They also do not spread in response to soluble LPS or to g-interferon (Figure 7F and 7G). The mutant is also significantly more resistant to *E. coli* than wild type (not shown).

Since a MDCK mutant selected for resistance to Concanavalin A is coincidentally resistant to ricin and to *P. aeruginosa* [47], we asked the inverse question of whether the RAW 264.7 mutant resists these lectins. When grown continuously in their presence, the mutant is at least 2x more resistant to both ConA and RCA I than wildtype (Table 2).

**Table 2:** Comparative Sensitivity of the Mutant and Wildtype Cells.

Clones	LD_50_ (µg/ml)		
	ConA	RCA	LPS
RAW	∼20	∼0.1	∼0.1
ResRAW	∼70	∼0.5	∼5

Sensitivity was evaluated by exposing cells to the lectins or to LPS continuously for 2–3 days.

#### Signaling

Since the mutant cells resist exposure to PAO1, they could have lesions at the level of TLR4 or downstream lesions along a signaling pathway. Nevertheless, as for wildtype cells, the p65 subunit of NF-kB relocates massively to the nucleus of the mutant cells within 1 h of exposure to PAO1 at MOI 10 (Figure 4A – panel 6). Relocation of p65 is also observed with soluble LPS (Figure 4A – panel 7). Consistent with the, Western blotting shows that the level of IkBα in the mutant decreases after exposure to PAO1 (Figure 4B).

Furthermore, exposure of the mutant to PAO1 strongly stimulates the release of several cytokines (Figure 5). In fact, the levels of secreted IL-6, MIP2 and TNF-α increase more rapidly than for wildtype. Thus, judging from both the relocation of p65 and from cytokine release, TLR4 signaling occurs in the mutant cells.

#### Transcriptional Profiles

The ability of the mutant cells to tolerate *P. aeruginosa* could reflect their steady-state phenotype or their differential hyper- or hypo-responsiveness when exposed to *P. aeruginosa*. We therefore compared the transcriptional profiles of these cells to wildtype, both before and after exposure. The following paragraphs summarize these observations.

#### Steady-state profiles (Tables S5C, S6I; S7(9i), S7(9d))

Before exposure to PAO1, the mutant has a transcriptional profile that differs from wildtype. Resistant cells show relative upregulation (10^−6^<p<10^−3^) of categories that are most enriched in signal transduction and prostaglandin biology. Specific transcripts encode

- The TGFb receptor,
- G-protein-coupled receptors for two bioactive lipids (platelet-activating factor, sphingosine phosphate),
- The receptor for programmed cell death ligand 1, and two semaphorin receptors (plexins). The semaphorins and corresponding receptors are best-characterized regarding neuronal migration and outgrowth of neuronal processes; however, they have also been implicated in inflammatory responses in conjunction with TGFb [48].
- Curiously, a GTPase-activating protein (Rgs1) that inhibits GPCR signaling is also upregulated.

The most strongly downregulated categories (10^−7^<p<10^−3)^ before exposure to PAO1 are immune and inflammatory responses. They include transcripts that encode one of the subunits that functions in conjunction with Tlr4 (CD14), as well as Tlr8.

Transcripts related to prostaglandin biology are either upregulated (Cox-1, Ptgds-2) or downregulated (Cox-2, protaglandin E4 receptor). A similar lack of uniformity is seen for proteins important for anti-viral responses (RNase L *vs* Ifitm3, Ifih1, Oas2).

#### Responses of resistant cells (1 h exposure: Tables S5D, S6E, S7(5i/d); 1+8 h exposure: Tables S5E, S6F, S7(6i/d))

When the mutant cells are exposed to *P. aeruginosa* for 1 h or for 1 h followed by 8 h, major transcriptional changes occur. As for wildtype, the following categories are most strongly upregulated (10^−16^<p<10^−6^) at both time points: apoptosis, immune response, inflammatory response, positive regulation of I-kB kinase-NF-kB cascade, anti-apoptosis.

At the 1 h time point, the most strongly downregulated categories (10^−16^<p<10^−6^) seem not to fall into coherent groupings. At 1+8 h, the categories that show strongest downregulation (10^−22^<p<10^−14^) are indicative of cell cycle arrest, as for wildtype. This similarity is notable since the resistant cells are destined to survive.

The progression of these responses through time is indicated in Tables S6C and S6D (wildtype) and S6G/H (mutant). To make this assessment, we have focused on transcripts whose levels do not change more than 2x in the 1 h *vs* 0 h tabulation. We then enumerate the intensity levels of the same group of transcripts in the tabulation of 1+8 h *vs* 0 h data for the same cell type.

For wildtype cells, transcripts that show >10x induction at 1+8 h (but not at 1 h) are Ccl2, Cd274, Edn1, Gbp1, Il1f6, Isg15, Rsad2, and Serpinb9. For the mutant, some responses are shared (Rsad2, Gbp1, Isg15); however, a distinctive new signature group of increases emerges. In this group, the recurrence of guanylate-binding proteins (Gbp3, Gbp5, Gbp6, as well as Gbp1) is striking. These proteins are also strongly induced by g-interferon, but their biological significance remains incompletely characterized [49]. Their induction by LPS is an order of magnitude less than induction by g-interferon and appears to be only transient [50]. Other proteins of possible importance for survival are Ifi202b, that promotes NF-kB signaling, and tenascin, that promotes cell rounding. No transcripts show strong downregulation when the 1+8 h data for resistant cells are compared to 1 h (Table S6H).

#### Wildtype vs resistant cell comparisons: (1 h exposure: (Tables S6J/J′; 1+8 h exposure: Tables S6K/K′)

The distinctive characteristics of a sensitive cell could be either positive or negative by comparison to a resistant cell. The top-to-bottom order of entries in Table S3 displays a) increases seen in the resistant cells after 1+8 h, that do not occur after 1+8 h in wildtype, b) increases seen in the wt after 1+8 h, that do not occur after 1+8 h in resistant cells, c) base-line comparison of mutant cells to wildtype, and d) transcripts thought to be relevant to survival (<0.5x or >2.0x).

In the 1+8 h samples for the mutant, points of particular interest are summarized in Table S4. It includes

- Transcripts that are expected to promote TLR signaling, including CD14, If202b, Nfkb1 and Traf5, that increase, and Nfkbie and Gpr109a, that decrease. TLR signaling could also be reduced by the increase in Tnip and the decrease in Tlr4 and Tlr13.
- Transcripts that are expected to promote JAK/STAT signaling, including Grap and Nfam, that increase, and Socs3 that decreases. JAK/STAT signaling could also be reduced by the decrease of JAK2 and the spingosine-phosphate receptor, S1pr1, that sustains SOX3 signaling.
- Transcripts that are expected to promote GPCR signaling, including E2f8, that increases, and Rgs1 and Trem2, that decrease.
- Transcripts that are expected to promote growth, including Gbp3, Gbp5, Gbp6, Myc, Plk2, Pkrir and Rasgrp3, that increase, and the decrease of CD274 (the receptor for the programmed cell death ligand), Fas, Phlda1 and Serpin b9. Growth could be opposed by the relative increase of Wee1.

Although the mutant cells survive, they show many of the initial changes that are also characteristic of the wildtype, e.g. major downregulation of histone transcripts. One might expect that resistance results from dampening of the initial changes. Nevertheless, only very few transcripts that are upregulated in wildtype at both +1 h and 1+8 h are upregulated in the mutant only at the initial time point. These are the transcription factor, Bhlbhb2, and Ifrd1.

## Discussion

The encounter between *P. aeruginosa* and host cells in the lung is seldom catastrophic unless the host is compromised. The case of cystic fibrosis (CF) is striking, in that *P. aeruginosa* infections account for a large fraction of CF deaths [6]. Several hypotheses can account for the exaggerated inflammatory responses of cells of CF patients: 1) that binding of bacterial LPS to cell surface CFTR normally elicits protective responses, e.g. phagocytosis and rapid initiation of signaling mediated by IL-1 [12], [23], 2) that alterations of glycosylation that are characteristic of the surfaces of CF cells sensitize them to *P. aeruginosa*, e.g. [6], or 3) that alteration of the composition of airway surface liquid by CFTR activity affects bacterial clearance. Given this lack of unanimity, additional approaches will be needed to learn how to make cells resistant to *P. aeruginosa*.

This study aims to identify especially sensitive features of the target cell, rather than describing all possible means by which the bacterium could kill this target. For this reason, we have abbreviated the time of exposure to pathogen and used a low multiplicity of infection. As for many cell types in CF, the target cells that we have chosen (RAW 264.7 monocyte/macrophages) do not express the cystic fibrosis transmembrane regulator. We surprisingly observe that several classical mechanisms of toxicity are irrelevant: Neither type II and type III secretion, pili nor flagella are required. Although cell contact is required, the only way to implicate LPS in toxicity is with the proviso that it must be in concentrated form, i.e. soluble LPS is toxic but on a molar basis it is orders of magnitude less toxic than intact *P. aeruginosa*.

Since *P. aeruginosa* seems not to be phagocytosed under the conditions used, a powerful signaling cascade(s) is presumably initiated upon contact with the cell surface. This same pathway could be responsible for initiating translocation of the p65 subunit of NF-kB into the nucleus and eliciting cytokine secretion. Intriguingly, toxicity is not accompanied by caspase 3 activation and inhibition of this enzyme does not block toxicity, although apoptosis can result from the interaction of *P. aeruginosa* with other cell targets [51], [52]. Thus, in agreement with observations of some other host-pathogen interactions, death does not require apoptosis [53]–[55]. Judging from the impact of a JNK inhibitor, this kinase may be central to the toxicity of PAO1 [56], [57].

**Figure 8:**
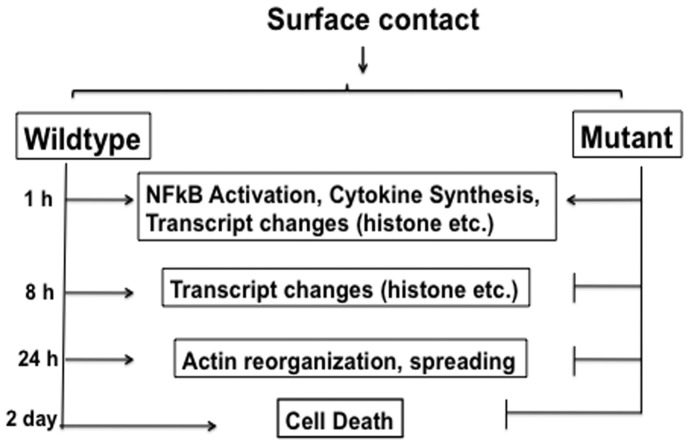
Overeview of the responses of wildtype and mutant cells. As is detailed in the text, the present observations show that both cell types exhibit comparable initial changes, including NF-kB activation, cytokine secretion, and downregulation of transcripts that are essential for continued growth. Nevertheless, only the wildtype cells are ultimately killed. The mutant cells show distinct upregulation of transcripts that are associated with responses to g-interferon. These changes could be of central importance for survival.

To identify functionally significant intermediates, we generated cells that resist the toxicity of *P. aeruginosa*. As summarized in Figure 8, they show some but not all of the responses that are characteristic of wildtype cells. Strikingly, these cells do not show morphological signs of activation (spreading); however, they do respond strongly to PAO1. In fact, they are hyper-responsive in the sense that PAO1 elicits cytokine secretion and selected transcriptional changes even more dramatically than for wildtype. Over-reaction could be part of a protective response due either to induction of protective factors or to repression of factors that normally oppose activation. Analysis of the possible mechanism(s) of toxicity is simplified by the demonstration that factors released from wildtype target cells and bacterial supernatants are not themselves toxic. Moreover, supernatants from stimulated mutant cells do not protect naïve wildtype cells. We conclude that, although corresponding autocrine loops surely exist, they are not key to toxicity.

Although cells resistant to *E. coli* LPS have been studied previously, we are not aware of studies that have used live *P. aeruginosa* for selection. Among LPS-resistant cells are

- Cells that cannot signal through TLR4: A J774.1 macrophage mutant [58], and a CHO cell mutant that has a defect in the LPS co-receptor, MD-2 [59].
- Cells that bind LPS: A WEHI-3 macrophage-derived mutant [60], and a CHO cell mutant that shows normal translocation of NF-kB upon LPS treatment [61]. Like the CHO cell mutant, the new mutants are still responsive to PAO1.

Our transcription profile data provide a point of reference for understanding the sensitivity/resistance of both the normal RAW 264.7 cell and the mutant. These stable phenotypic differences could result from multiple ramifications of a single genetic change, from a widespread epigenetic change, or from chromosomal rearrangements.

The differences of steady state profiles of mutant *vs* wildtype before stimulation suggest that the mutant is well-prepared to participate in several types of signaling: multiple receptors are upregulated (for TGF, lipid mediators, semaphorins). Moreover, although they do respond to *P. aeruginosa*, the titer of two Tlr receptors (CD14, Tlr8) is reduced. They also exhibit a reduced level of at least one pro-apoptotic protein (Ifi27).

When wildtype cells are stimulated for 1 h, they already show strong proinflammatory responses that persist through time, along with dramatic down-regulation of housekeeping genes (encoding histones, ribosomal proteins, etc.) and selected receptors. These changes provide a molecular signature of the non-apoptotic form of toxicity that is triggered in this situation. Very comparable changes occur in the mutant, but by the 1+8 h time point, several novel increases occur. The strongest of these encode several of the “guanylate-binding proteins,” tenascin, that promotes spreading, and Ifi202b, that promotes NF-kB signaling. Also striking is the upregulation of several growth-promoting transcripts (Myc, Rasgpr3, Plk2, Pkrir) along with downregulation of several that could oppose survival (CD274, Fas, Phlda1, Serpin b9). Taken together, these observations suggest the value of preliminary expression of multiple receptors, and induction of a cluster of powerful growth-promoting factors (Figure 8).

Interestingly, there is a very striking enrichment of a repertoire of g-interferon-inducible transcripts among the responses that are stronger in the mutant than in wildtype – Table S3. As g-interferon transcripts do not increase, these observations could signify that the mutant allows extensive input into g-interferon signaling pathways. Nevertheless, we do not observe upregulation of transcription factors (e.g. DRAF, VAF, VIF) that mediate the induction of subsets of g-interferon targets, either by g-interferon itself or by dsRNA [62]. Perhaps the induced repertoire results from “cross-talk” between signaling pathways. “Cross-talk” has been detected under other circumstances, with emphasis on g-interferon's non-classical stimulation of pathways other than those normally under control of STAT1 [63], [64].

Few transcription factors appear to be up- or down-regulated in the mutant and those that are affected are not obviously linked to g-interferon. Thus, the widely-expressed factor, bhlbh2, is increased in the mutant before exposure to *P. aeruginosa*, while bhlbh3 [65] is selectively reduced in the mutant after stimulation. E2f8 is selectively reduced after stimulation only in wildtype. Other possible contributors are kinases that impact STAT proteins or their regulators, e.g. Socs proteins.

g-interferon signals principally through JAK1, JAK2 and STAT1. Nevertheless, g-interferon signaling can also lead to STAT-independent activation of ERK1/2 and PI3-kinase, e.g. [63].

There has been no agreement as to whether g-interferon can protect cells against *P. aeruginosa* [66], [67] perhaps because one of the secretory proteases of this organism can degrade g-interferon [68]. The present observations reopen this issue and forcefully raise the question of whether a subset of g-interferon-responsive proteins effectively opposes *P. aeruginosa* [69].

The ease with which resistant cells can be obtained suggests that comparable shifts of transcriptional specificity could normally enable wildtype cells in the host organism to resist *P. aeruginosa*. Identification of factors that mediate these shifts could therefore help identify molecular targets of therapeutic value.

g-interferon-induced proteins are often critical for responses to intracellular pathogens; however, evidence for the importance of Gbp's in defense against viral and bacterial infections remains fragmentary. Moreover, although *Listeria monocytogenes* and *Toxoplasma gondii* upregulate multiple GBPs *in vivo*, the toxicity of *Salmonella* (toward RAW 264.7 cells) is actually potentiated by GBP-5 [44], [70]. Other studies have shown that increased Gbp expression can inhibit cell growth and spreading, metalloprotease production and angiogenesis [71]–[74]. The present data set therefore presents a challenge for interpretation. One or more of the Gbp's - or other g-interferon-responsive proteins - could account for the resistance of the mutant cells.

## Materials and Methods

### Source of reagents

Except where indicated otherwise, all chemicals were from Sigma-Aldrich Chemical Co.

### Bacterial Strains and Growth Conditions

Strains and plasmids used are listed in Table S1. The strain of PAO1 is proficient in Type I, II and III secretion (Dr. A. Rietsch, personal communication). Bacteria were routinely grown overnight with shaking at 37°C in Luria-Bertani medium. The concentration of bacteria was determined by spectrophotometry (OD_600_ of 0.1 was equivalent to 1×10^8^ cfu/ml). The growth of the bacteria could be completely inhibited with a mixture of 200 µg/ml of gentamicin (Sigma-Aldrich, G-1914) and 200 µg/ml streptomycin (Sigma-Aldrich, S-6501).

### Cell Culture

RAW 264.7 (ATCC T1B-71), a mouse monocyte-derived cell line, was grown in DMEM (Invitrogen, Carlsbad, CA), supplemented with 10% heat inactivated fetal bovine serum (Invitrogen), 5% CO_2_ at 37°C. Cells were seeded at 1×10^4^ cells/500 µl on coverslips (for microscopy) or 1×10^5^ cells/well in 12 well plates (for MTT assays) or 2×10^5^ cells/well in 6 well plates (for other assays). For selected experiments, 1×10^6^ cells were transduced with a hrGFP retroviral supernatant (Stratagene) and cells expressing green fluorescent protein (GFP) were cloned by limiting dilution.

### Exposure to *P. aeruginosa*

*P. aeruginosa* were grown overnight, diluted and regrown for another 2 h to an OD_600_ of 0.1 so that they were in exponential growth phase. Cultures were centrifuged at 10,000 rpm for 10 min and were resuspended in tissue culture medium. *P. aeruginosa* was added to RAW 264.7 cells in their serum-containing growth medium at a MOI of 10∶1 (if not mentioned otherwise). After 1 h, the cells were washed 3× with PBS and fresh medium containing gentamicin (200 µg/ml) and streptomycin (200 µg/ml) was added to kill extracellular bacteria during continued culture. To estimate the titer of internalized bacteria, we prepared cell lysates according to [28] and plated them on LB plates for quantitation.

### Mutant Selection

5×10^5^ RAW 264.7 cells were exposed to PAO1 at MOI = 5 for 1 h in medium without antibiotics at 37°C. Cells were washed with PBS and received fresh medium supplemented with 200 µg/ml gentamicin and 200 µg/ml streptomycin. The surviving cells (viability 5–10%) were allowed to grow for 3–5 days until viability was again excellent. The cells were then split and again exposed to *P. aeruginosa*. This process was iterated 10×. Both naïve and cycled RAW 264.7 cells were cloned by limiting dilution.

### Cytotoxicity Assays

Cytotoxicity was determined quantitatively using a 3-[4,5-Dimethylthiazol-2-yl]-2,5-diphenyltetrazolium bromide (MTT) (Invitrogen, M-6494) cleavage assay (Mosmann, 1983). For this assay, 1×10^5^ cells were plated onto 12-well plates 1 day prior to the experiment and exposed to bacteria as above. After removal of the supernatant the cells were rinsed with PBS. At this point, 100 µl of 5 mg/ml MTT in PBS was added per ml of culture media and incubated for 1 h at 37°C. The supernatant was discarded and 1 ml of stop solution (20% SDS, 50% dimethylformamide) was added to each well to dissolve the formazone crystals. Absorbance was recorded at 570 nm. Readings taken for uninfected control cells were used for normalization. The percent viability was calculated by the formula (*A*_570 nm_)_sample_/(*A*_570 nm_)_control_×100. The controls were not exposed to bacteria. For these and all other quantitative assays, standard deviations were calculated and illustrated as error bars.

### Statistical analysis

Statistical analysis was performed using an unpaired *t* test (Prism; GraphPad Software). *p* values less than 0.05 were considered significant.

### Preparation of culture supernatants

To ascertain whether toxicity was due to soluble factors released by bacteria or RAW 264.7 cells, several experiments were performed. To investigate the toxicity of bacterial supernatants, two procedures were used: 1) graded amount of bacterial culture were centrifuged for 5 min at 10,000 rpm. The pellets were discarded and the supernatants were passed through 0.22 µm filters (Millipore), mixed 1∶10 with growth medium and added to RAW 264.7 cultures (2.5×10^5^ cells/well) for 1 h. Control plates received the same volume of LB medium. 2) PAO1 was added at MOI = 10 and 50 to culture wells either with or lacking RAW cells. After 1 h, the resulting supernatants were recovered, centrifuged for 5 min at 10,000 rpm and filtered through 0.22 µm filters. They were then added (without dilution) to naïve RAW 264.7 cultures. After incubation for 1 h at 37°C, they were transferred to fresh medium for two days for examination and MTT assay.

### Microscopy

RAW 264.7 cells (5×10^3^) were grown overnight on 12 mm glass coverslips and exposed to bacteria at MOI = 10 and 50 at 37°C. After 1 h, the cells were washed with PBS and fixed with 4% paraformaldehyde in PBS for 15 min at room temperature, washed with PBS, incubated with 0.1 M glycine in PBS for 5 min followed by 0.1% (v/v) Triton X-100 in PBS for 1 min at room temperature. They were then stained with 0.15 µM phalloidin in PBS (Sigma, P-1951) for 15 min at room temperature in the dark and washed with PBS. The coverslips were then stained with 0.015 µM 4,6-diamidino-2-phenylindole (DAPI) (Sigma-Aldrich, D-9542) in PBS for 2 min in the dark, washed with PBS, and mounted on glass slides using gel mount antifade reagent (Biomeda Corp.). The slides were viewed at 100× magnification under oil immersion in an epifluorescence microscope and by Deltavision microscopy.

### p65 Translocation Assays

For indirect immunofluorescence analysis, 5×10^3^ cells were grown on 18 mm glass coverslips and exposed to either PAO1, MOI = 10 or to 100 µg/ml LPS of *P. aeruginosa*, serotype 10 (Sigma-Aldrich L-9143), for 1 h, washed, and recultured as required. Cells were fixed with 4% paraformaldehyde in PBS for 15 min at room temperature, permeabilized with 0.1% Triton X-100 in PBS for 1 min at room temperature and stained with rabbit anti-p65 (1∶100; eBioscience Ltd) in PBS containing 10% goat serum for 30 min at room temperature. Cells were incubated with Lissamine-Rhodamine conjugated goat anti-rabbit IgG (Jackson ImmunoResearch Laboratories) in PBS at room temperature for 30 min and washed 3× with PBS. Nuclei were stained using DAPI as above, washed, mounted and examined. For detecting activation of the cells, the cells were treated with LPS or PAO1 as described, fixed and viewed at 40× in phase contrast microscope.

### Apoptosis Assays

#### DNA Fragmentation

RAW 264.7 cells were plated in six well plates (1×10^6^ cells/well) and were exposed to PAO1, MOI = 10 for 1 h. The cells were then washed 3× in PBS and recultured. After 1–24 h the cells were collected by centrifugation and lysed with 200 µl of lysis buffer (10 mM Tris-Cl, pH 8.0, 10 mM EDTA and 0.05% Triton X-100) [75]. Lysates were incubated with 1 µg/ml of RNaseA, followed by 0.5 µg/ml of Proteinase K for 1 h at 37°C and 50°C respectively. DNA was extracted using Tris-saturated phenol, pH 7.4, chloroform and isoamyl alcohol (25∶24∶1) and precipitated with isopropyl alcohol at −20°C. The precipitate was centrifuged at 12,000×g for 15 min and the pellet was air dried and suspended in 10 mM Tris-Cl, pH 8.0. Samples of DNA (2.5 µg) were electrophoresed in 1.2% agarose gels in TAE, and stained with ethidium bromide. As a positive control, cells were also treated with staurosporine (1 µg/ml for 2 h), a kinase inhibitor that induces apoptosis [76], and DNA samples were processed exactly as above.

### Caspase-3 Assay

Cells in six well plates (1×10^6^ cells/well) were exposed to PAO1 and samples were collected at different time points by centrifugation at 1,000×g for 15 min. The pelleted cells were lysed in 100 µl lysis buffer (250 mM HEPES, pH 7.4; 25 mM CHAPS and 25 mM DTT), incubated on ice for 30 min and then centrifuged at 12,000×g for 15 min. Supernatants were incubated with the caspase-3 substrate, 0.2 mM Ac-DEVD-pNA (Sigma-Aldrich) overnight at 37°C. Product formation was measured spectrophotometricaly at 405 nm. As controls, the cells were pretreated for 4 h with 0.02 mM cell-permeable inhibitor of caspase-3 (Ac-DEVD-CHO) prior to exposure to bacteria. The inhibitors were also present after the cells were washed free of bacteria.

### Western Blot Analysis

Both wildtype and mutant cells were exposed (or not) to PAO1 as mentioned above. The cells were washed 3× in PBS, and lysed using cold 1× lysis buffer (Cell Signaling Technology, Beverly, MA). Total protein was quantified using standard BCA assay, and 10 µg of protein were separated by 12% SDS-polyacrylamide gel, transferred to nitrocellulose membrane, and probed for total IkB and ß-actin (Cell Signaling Technology). Proteins were detected using HRP-conjugated secondary antibodies and developed with Supersignal West Femto Maximum Sensitivity Substrate (Pierce).

### Cytokine Measurements

To quantify cytokine secretion, cells were grown overnight in a 12 well plate (1×10^5^ cells/well) and exposed to PAO1 at MOI = 10 for 1 h. The cells were then recultured. Supernatants were collected after 0, 1, 6 and 24 h to measure released cytokines. The Mouse Cytokine FMAP BASE kit (R&D Bioscience) was used to quantitate interleukins IL-6, MIP-2 and TNF-α. For this purpose, 100 µl of the supernatants were added to 96 well microtiter plates containing antibody-coated bead complexes. Plates were analyzed using the Luminex 100 analyzer, version 7.1 (Luminex Corp., Austin, TX). A minimum of 100 events (beads) was collected for each cytokine/sample and median fluorescence intensities were obtained. Cytokine concentrations were calculated based on standard curve data using STATLIA from Brendan version 3.2.

### Microarray Analysis

Triplicate cultures of RAW 264.7 cells and mutants were harvested 0, 1 and 8 h after 1 h exposure to PAO1, MOI = 10 for 1 h; control samples (0 h time point) were not exposed to bacteria. In each case cells were washed 3× with cold PBS and total RNA was isolated using RNeasy kit (QIAGEN) and then further processed by the Gene Expression and Genotyping Facility of the Case Comprehensive Cancer Center. Affymetrix analysis of ∼1 µg of RNA used mouse whole transcriptome arrays (Mouse GeneChip 1.0 ST chip) and Affymetrix software. Hybridization was conducted overnight at 45°C with agitation. Post-hybridization, washing and staining were performed in a Fluidics Station 450 (Affymetrix). Hybridization signal was measured using a GeneChip scanner (Affymetrix GC3000) to detect the fluorescence at each probe loci. Signal values were generated using Expression Console 1.1 (EC, Affymetrix). Signals were normalized using the RMA (Robust multichip Analysis) feature of EC. Fold changes in expression were filtered by means of a 1.5 fold change in a binary comparison ratio data between group means.

BAMarray analysis (incorporating Bayesian Analysis of Microarray data) was also used to analyze our data set. This software is a statistical tool which is capable of conferring significance upon observed differences between the means of individual gene expression signals of groups of samples. False detection rates get smaller with increasing numbers of replicates per group. The software is freely available to academic users. Data were exported as space-delimited text files which were imported into MS excel. Probset identifiers are accompanied by the following significance indices: “1” (significantly increased over the base line group); “0” (not significantly different from the baseline group) and “−1” (significantly decreased compared to the baseline group). BAM analysis deals only with statistical significance and does not comment on the magnitude of the fold change. We cross-referenced our gene change results from BAMarray with the 1.5 fold changes between group means describe above. This produced a keeper data set which we subsequently took into other software for pattern and pathway mining analyses.

Functional gene classifications were derived from Gene Ontology information found in National Center for Biotechnology Information (NCBI) database and from independent literature searches.

There are three sets of supplementary Tables (Tables S5, S6, S7), as inventoried end of the list of supplementary tables:

Tables S5A–S5B, S5D–S5E “Full Data Sets: Comparisons to t 0” provide the basic data sets in which the values for each condition (e.g. after 1 h exposure to *P. aeruginosa*) is compared to that of resting cells (t 0) of the same type. Table S5C compares the transcript levels for the resistant cells to wildtype cells.

In Tables S6A–K “Homotypic and Heterotypic Comparisons for Annotated Genes,” the data sets have been simplified by including only annotated genes, e.g. Table S6A is a simplified version of Table S5A. Tables S6A, S6B, S6E, S6F include comparative information, listing genes that change in one time interval but not more than 1.5× in another (e.g. in the wildtype t1+8 h vs t 0 comparison but not more than 1.5× in the wildtype t1 h vs t 0 comparison). Tables S6C and S6D (for wt cells, like S6G and S6H for the resistant cells) lists entries for which changes are seen at one time point (e.g. 1+8 h vs t0) but not at another (e.g. 1 h vs t 0). Table S6I (as well as S7(9i)/7(9d)) compares the transcriptome of resting wildtype cells with that of resting resistant cells. Tables S6J and S6K make comparisons between the wildtype and resistant cells. The comparisons designated (‘) are inverse to those lacking this designation, e.g. Table S6J is “changed in wildtype 1 h vs t 0 but not more than 1.5× in resistant cells 1 h vs t 0”, while Table S6J′ is “changed in resistant 1 h vs t 0 but not in wildtype 1 h vs t 0”. In the comparative tables, changes must be >1.5 x to be included.

Tables S7(1,2,5,6,9) - “Gene Ontology Groups” summarize gene ontology classifications and therefore include only annotated genes. An overview summary of these data is given in Tables 1 and S2. We have eliminated categories for which there was only a single entry. The suffixes (i) or (d) indicate whether increases or decreases are observed.

In these Tables, the columns specify probe set IDs, average values for one condition, average values for the second condition, the quotient of the two, the “posterior call” [that indicates whether, according to Bam analysis, the change is significant and is an increase (+1) or decrease (−1)], the gene symbol (if known) and gene description (if known). The columns specify the functional category, its type, the total number of genes within that category in the spread sheet, the expanded number of such genes, the number of genes in this analysis that are included in the category, the number expressed as a percent of genes in the category, the specific genes in question, the p-value for having this overlap, the data source for annotations, and the rank order of each entry. The successive spread sheets are either total, or successively trimmed by p-value, or trimmed to eliminate entries where there is only a single entry.

## Supporting Information

Figure S1 **Investigations of the importance of LPS.** **A**) A schematic of LPS structure, indicating the portions of the glycan that are absent in selected mutants. **B**) A two-day assay of the toxicity of *Pseudomonas* expressing truncated forms of LPS, by comparison to wt PAO1. **C**) Standard two-day assays show that polymyxin B blocks the toxicity of soluble LPS, but not intact PAO1.
(TIF)

Figure S2 **Studies of Murine Alveolar Macrophages.** **A**) MTT assays to quantitate cell number. Uninfected cells compared to cells exposed to *P. aeruginosa* and then recultured over 4 days. **B**) Annexin V binding evaluated by flow cytometry: Black: non-specific signal detected with an isotype-matched control antibody. Red: Cells not exposed to bacteria or to staurosporin. Blue: Cells exposed to bacteria or to staurosporin, as indicated. **C**) Caspase involvement, as in Figure 6B. Cells were exposed to PAO1 (MOI 10, 1 h) washed, and returned to culture. Caspase-3 activity was measured after 1–24 h (P1, P3 etc). Control cells not exposed to bacteria were collected at the same time points (C1, C3 etc.). Sample treated with staurosporine (STS, 1 µg/ml) with or without caspase-3 inhibitor (Inh) for 3 h were used as a positive control.
(TIF)

Methods S1 **Supplementary Methods.**
(DOC)

Table S1 **Bacterial strains and plasmids used in this study are listed in Table S1.**
(DOC)

Table S2 **Summary of Gene Ontology Information.**
(DOC)

Table S3 **Comparison of wildtype and resistant cell responses.**
(DOC)

Table S4 **Summary of Relative Changes After 1+8 h Exposure to** ***Pseudomonas aeruginosa*** **.**
(DOC)

Table S5 **Full Data Sets: Comparisons to t0 - Tables S5A–S5E.** Table S5A: WT t1 hr vs WT t0. Table S5B: WT t1+8 h vs WT t0. Table S5C: Resistant t0 vs WT t0. Table S5D: Resistant t1 hr vs t0. Table S5E: Resistant t1+8 hr vs t0.
(ZIP)

Table S6 **Homotypic and Heterotypic Comparisons for Annotated Genes - Tables S6A–S6K.** Table S6A: Changes in WT t1 vs WT t0. Table S6B: Changes in WT t1+8 vs WT t0. Table S6C: Changes in WT t1+8 vs WT t0, not in WT t1 vs t0. Table S6D: Changes in WT t1 vs WT t0, not in WT t1+8 vs t0. Table S6E: Changes in resistant t1 vs t0. Table S6F: Changes in resistant t1+8 vs t0. Table S6G: Changes in resistant t1+8 vs t0, not in resistant t1 vs t0. Table S6H: Changes in resistant t1 vs t0, not in resistant t1+8 vs t0. Table S6I: Changes in resistant t0 vs WT t0. Table S6J: Changes in WT t1 vs WT t0, not in resistant t1 vs t0. Table S6J′: Changes in resistant t1 vs t0, not in WT t1 vs t0. Table S6K: Changes in WT t1+8 vs WT t0, not in resistant t1+8 vs t0. Table S6K′: Changes in resistant t1+8 vs t0, not in WT t1+8 vs t0.
(ZIP)

Table S7 **Gene Ontology Groups – S7(1)–S7(9).** Table S7(1d): Groups associated with WT t1 vs WT t0 decreases. Table S7(1i): Groups associated with WT t1 vs WT t0 increases. Table S7(2d): Groups associated with WT t1+8 vs WT t0 decreases. Table S7(2i): Groups associated with WT t1+8 vs WT t0 increases. Table S7(5d): Groups associated with resistant t1 vs resistant t0 decreases. Table S7(5i): Groups associated with resistant t1 vs resistant t0 increases. Table S7(6d): Groups associated with resistant t1+8 vs resistant t0 decreases. Table S7(6i): Groups associated with resistant t1+8 vs resistant t0 increases. Table S7(9d): Groups associated with resistant t0 vs WT t0 decreases. Table S7(9i): Groups associated with resistant t0 vs WT t0 increases.
(ZIP)
