# Supplementary figures and images for: Non-Apoptotic Toxicity of Pseudomonas aeruginosa toward Murine Cells
Source: PLoS One. 2013 Jan 24;8(1):e54245. doi: 10.1371/journal.pone.0054245 (PMC3554662; doi:10.1371/journal.pone.0054245)

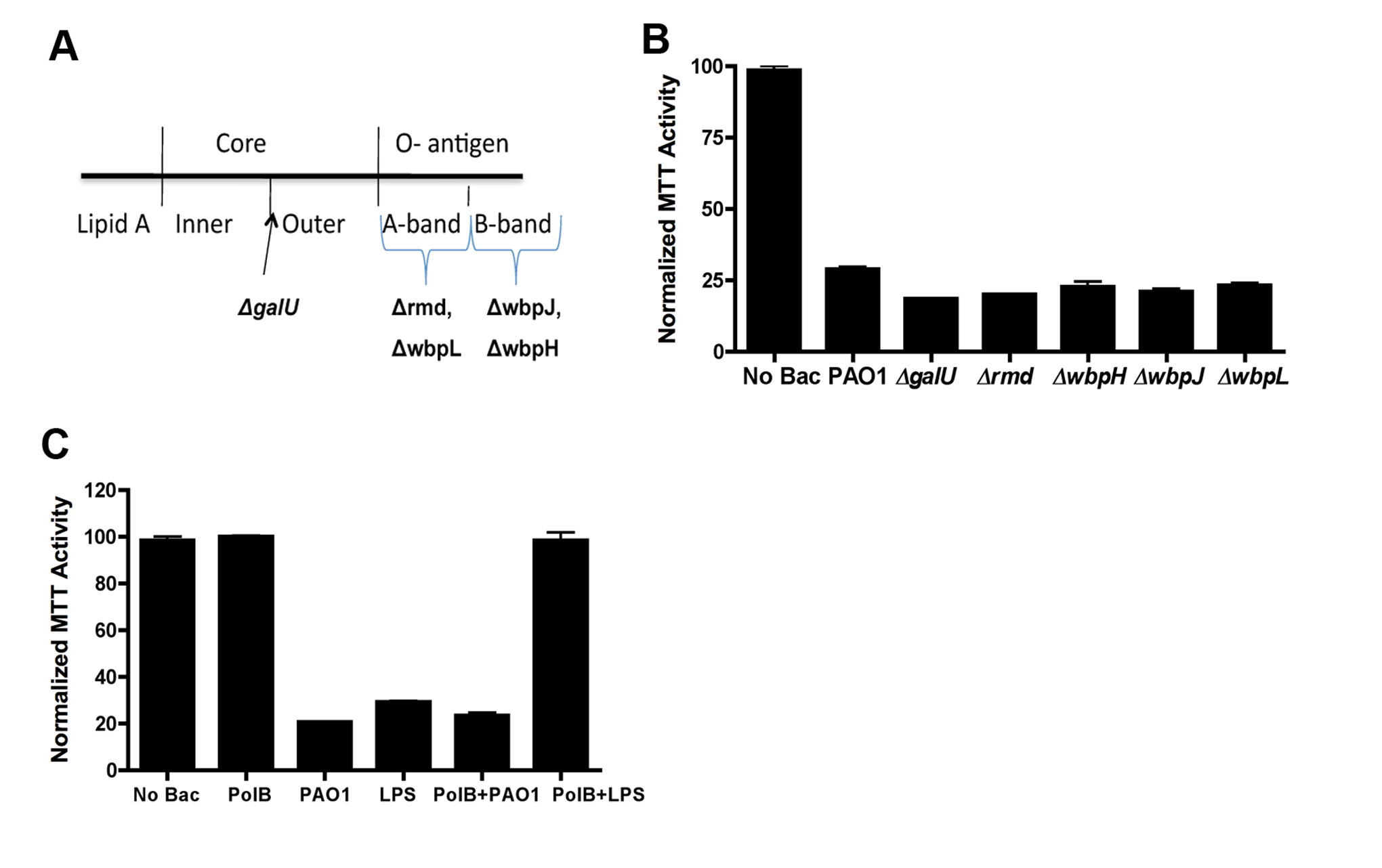

Supplement: Figure S1 — Investigations of the importance of LPS. A) A schematic of LPS structure, indicating the portions of the glycan that are absent in selected mutants. B) A two-day assay of the toxicity of Pseudomonas expressing truncated forms of LPS, by comparison to wt PAO1. C) Standard two-day assays show that polymyxin B blocks the toxicity of soluble LPS, but not intact PAO1. (TIF) [file pone.0054245.s001.tif]

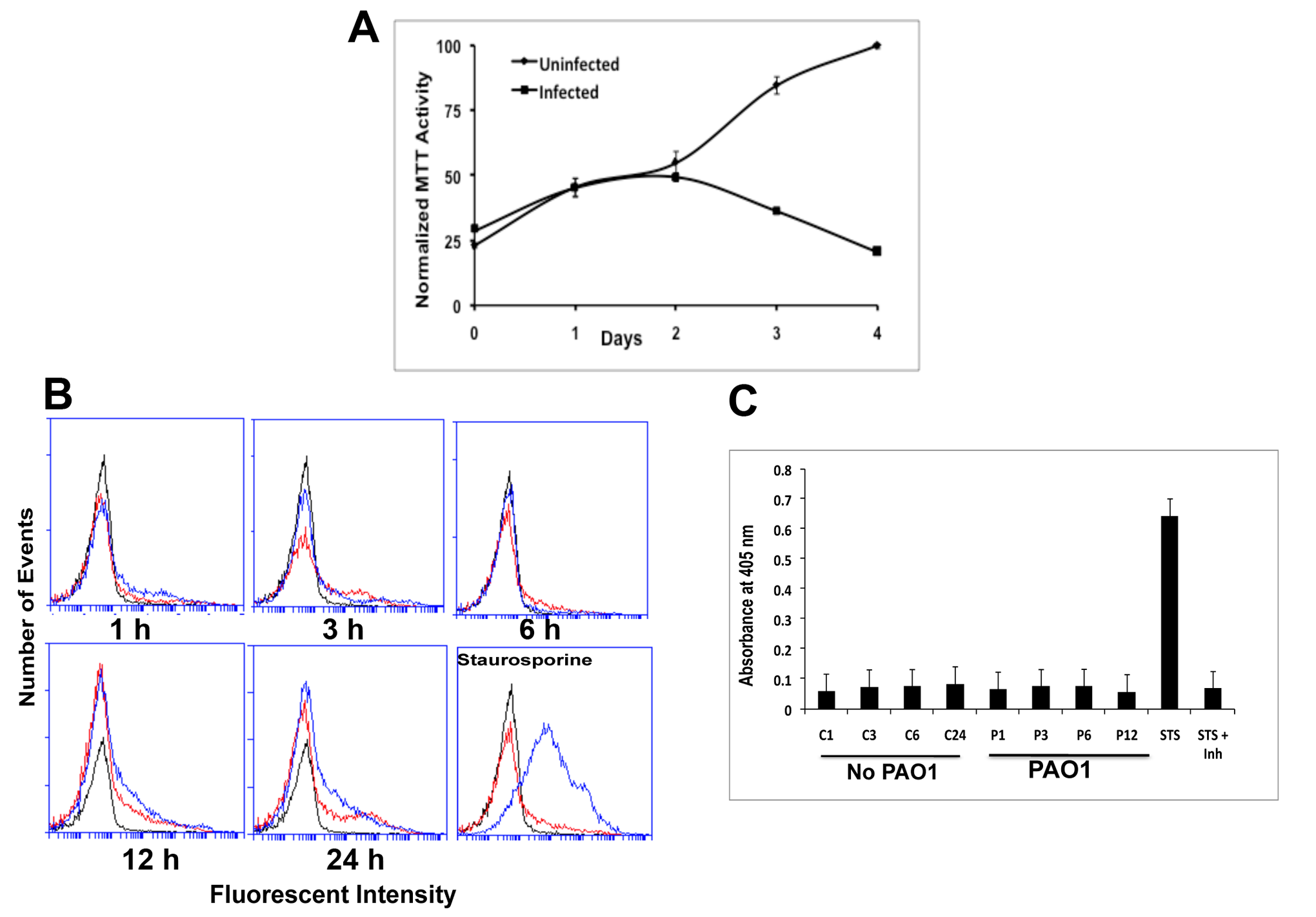

Supplement: Figure S2 — Studies of Murine Alveolar Macrophages. A) MTT assays to quantitate cell number. Uninfected cells compared to cells exposed to P. aeruginosa and then recultured over 4 days. B) Annexin V binding evaluated by flow cytometry: Black: non-specific signal detected with an isotype-matched control antibody. Red: Cells not exposed to bacteria or to staurosporin. Blue: Cells exposed to bacteria or to staurosporin, as indicated. C) Caspase involvement, as in Figure 6B. Cells were exposed to PAO1 (MOI 10, 1 h) washed, and returned to culture. Caspase-3 activity was measured after 1–24 h (P1, P3 etc). Control cells not exposed to bacteria were collected at the same time points (C1, C3 etc.). Sample treated with staurosporine (STS, 1 µg/ml) with or without caspase-3 inhibitor (Inh) for 3 h were used as a positive control. (TIF) [file pone.0054245.s002.tif]
