# Supplementary material for: Non-Apoptotic Toxicity of Pseudomonas aeruginosa toward Murine Cells
Source: PLoS One. 2013 Jan 24;8(1):e54245. doi: 10.1371/journal.pone.0054245 (PMC3554662; doi:10.1371/journal.pone.0054245)
Supplement: Methods S1 — Supplementary Methods. (DOC) [file pone.0054245.s003.doc]

**Supplementary Methods:**

**Mice, Alveolar Cell Harvest**

Animals were housed and cared for in fully accredited (AAALAC) animal facilities.  Mice were euthanized by overdose with xylazine and ketamine, as approved by the Panel on Euthanasia of the American Veterinary Medical Association.  Alveolar macrophages were collected from 6-8 weeks Balb/c old mice and cultured as described [37].

**Annexin V Staining**

Lung macrophages grown in six-well plates (1 x 10^6^ cells/well) were exposed to PAO1 and cells were collected at different time points. The resulting cell suspensions were filtered, washed with FACS buffer (1X PBS, 1% fetal bovine serum, 0.1% sodium azide) and single cell suspensions were obtained. Cells were then incubated with Fc blocking Ab (anti-mouse CD16/32; eBioscience) for 10 min on ice. To determine the percentage of apoptotic cells, cells were washed and stained with anti-mouse Annexin V antibody (eBiosciences). The cells were then washed in FACS buffer, fixed in 1% paraformaldehyde and analyzed by C6 Accuri flow cytometer.
