# Supplementary material for: Non-Apoptotic Toxicity of Pseudomonas aeruginosa toward Murine Cells
Source: PLoS One. 2013 Jan 24;8(1):e54245. doi: 10.1371/journal.pone.0054245 (PMC3554662; doi:10.1371/journal.pone.0054245)
Supplement: Table S1 — Bacterial strains and plasmids used in this study are listed in Table S1. (DOC) [file pone.0054245.s004.doc]

| **Strains or plasmid** | **Relevant genotype or phenotype** | **Source or References** |
| --- | --- | --- |
| Strains  PAO1 | Wild type | Dr. A. Rietsch |
| PAO1 *Δxcp* | deletion of the *xcp* gene | Dr. A. Rietsch |
| PAO1 *ΔpscC* | deletion of the psc*C* gene | Dr. A. Rietsch |
| PAO1 *ΔFliQ* | deletion of the *fliQ* gene | Dr. A. Rietsch |
| PAO1 *ΔFliC* | deletion of the *fliC* gene | Dr. A. Rietsch |
| PAO1 *ΔPilA* | deletion of the *pilA* gene | Dr. A. Rietsch |
| PAO1 *ΔgalU* | Complete inner core, but no outer core sugars | Dr. J. Lam |
| PAO1 *Δrmd* | Knockout of GDP-4-keto-6-deoxy-D-mannose reductase | Dr. J. Lam |
| PAO1 *ΔwbpJ* | deletion of the putative glycosyltransferase gene | Dr. G. Pier |
| PAO1 *ΔwbpH* | deletion of glycosyltransferase gene | Dr. G. Pier |
| PAO1 *ΔwbpL* | deletion of glycosyltransferase gene | Dr. G. Pier |
| Plasmids |  |  |
| pSMC21 | pUCP-based plasmid containing *gfpmut2*, Ap^r^, Cb^r^, Km^r^ | Dr. G. Toole |

**Table S1: Bacterial strains and plasmids used in this study**
