# Supplementary material for: Non-Apoptotic Toxicity of Pseudomonas aeruginosa toward Murine Cells
Source: PLoS One. 2013 Jan 24;8(1):e54245. doi: 10.1371/journal.pone.0054245 (PMC3554662; doi:10.1371/journal.pone.0054245)
Supplement: Table S2 — Summary of Gene Ontology Information. (DOC) [file pone.0054245.s005.doc]

**Table S2: Summary of Gene Ontology Information**

| **1^o^ Data Table** | **Table trimmed** | **GO Table** | **Comparison** |  | **# genes** | **Lowest p value** | **Categories** (ordered by p-value – the numbers in parenthesis indicate the rank order of each category) |
| --- | --- | --- | --- | --- | --- | --- | --- |
| A1 | B1i | C1i | Wt 1 vs wt 0 | incr | 239 | 2.9 E-17 | Inflammatory response (3)  Immune response (4)  LPS responses (9)  Response to cytokine stimulus (13) |
|  | B1d | C1d |  | decr | 30** | 1.2 E-4** | Corticosterone response (2: CCND1 (G1/S cyclin), FOS)  Progesterone responses (3: FOS, TXNIP (thioredoxin-interacting protein) |
| A2 | B2i | C2i | Wt 1+8 vs wt 0 | incr | 294 | 2.7 E-25 | Immune response (1)  Inflammatory response (2)  LPS responses (11)  Response to cytokine stimulus (15) |
|  | B2d | C2d |  | decr | 127* | 1.5 E-36 | Cell cycle (1)  Cell division (2)  Mitosis (3)  Cell cycle regulation (4)  Nucleosome assembly (5)  Response to DNA damage (6)  Cell proliferation (7)  DNA repair (8)  DNA replication (9)  rRNA processing (10)  Regulation of cell cycle (11)  Response to corticosterone (12) |
|  | B3 |  | Wt 1+8 not wt 1 |  | 760 (134+, 456-) |  | Inhibited: Many histones, mitochondrial ribosomal proteins, cyclins, kinesins |
|  | B4 |  | Wt 1 not wt 1+8 |  | 179 (85+, 13-) |  |  |
|  | | | | | | | |
| A4 | B5i | C5i | Res 1 vs res 0 | incr | 165 | 3.3 E-16 | Inflammatory response (2)  Immune response (3)  LPS response (49)  Response to cytokine stimulus (154) |
|  | B5d | C5d |  | decr | 35** | 2.2 E-7** | Progesterone responses (1)  Corticosterone response (4) |
| A5 | B6i | C6i | Res 1+8 vs res 0 | incr | 176 | 2.5 E-23 | Inflammatory response (1)  Immune response (2)  LPS response (51)  Response to cytokine stimulus (119) |
|  | B6d | C6d |  | decr | 43 | 1.5 E-22 | Mitosis (1)  Cell division (2)  Cell cycle (3)  Nucleosome assembly (4)  Mitotic spindle organization (5)  Positive regulation of cell proliferation (10)  Cell cycle regulation (15)  DNA replication (16)  DNA repair (43) |
|  | B8 |  | Res 1+8 not res 1 |  | 197 (54+, 108-) |  | Fewer cyclins and histones than for wt |
|  | B7 |  | Res 1 not res 1+8 |  | 54 (33+, 4-) |  |  |
|  | | | | | | | |
| A3 | B9i | C9i | Res 0 vs wt 0 | incr | 30 | 3.2 E-5 | PGE synthesis (2, 4: PTGS1, PGDS)  Response to hypoxia (10: Cd24a, PLAU (plasminogen activator), TGFBR1)  Response to stress (13: NDRG4 (regulates signaling), GPR132 (a GPCR), TP53I11)  Inflammatory response (17: PTAFR (platelet activating factor R – leads to prostaglandin synthesis), CD97 (a GPCR), NDST1 (heparan sulfate deacetylase/sulfotransferase)  Immune response (19: CD86 (T cell activation), PTAFR, CD97, RGS1 (attenuates GPCR signaling)  Response to oxidative stress (23: PTGS1, DUSP1 (dephos MAPK))  Response to organic cyclic substance (24: TGFBR1, CD83)  Defense response (28: CD48, CD83) |
|  | B9d | C9d |  | decr | 73 | 1.2 E-7 | Immune response (1: CLEC4D (C-lectin-like), CD14, CXCL10 (induced by IFNg), CEBPB, IFIH1 (helicase induced by IFNb), PTGER4 (PGE4R), C5AR1, TLR8, FYB, IFITM3 (induced by IFNa, g), DHX58 (neg reg of innate resp), OAS2 (IFN induced anti-viral), PXDN (a peroxidase))  Inflammatory response (2: PTGS2 (=COX2), CD44, CD14, LYZ, CXCL10, CEBPB, CYBB (cyt B), TLR8) |
|  | B10 |  | Wt 1 vs 0, not res 1 vs 0 |  | 423 (135+, 33-) |  |  |
|  | B10’ |  | Res 1 vs 0, not wt 1 vs 0 |  | 40 (18+, 8-) |  | Increased: C5AR1, CD14, CD80, CDKinhibitor, Lima (LIM domain and actin binding)  Decreased: (sphingosine-P R) |
|  | B11 |  | Wt 1+8 vs 0, not res 1+8 vs 0 |  | 945 (186+, 424-) |  | Wt losses: 13 mitochondrial ATP synthase subunits, 6 cyclins, 13 histones, 8 Mribosomal proteins, 4 NADH dehydrogenase subunits, 11 subunits of DNA or RNA polymerases, selected ribosomal proteins, Arp 2/3 subunit. etc;  Wt increases: Socs3 (suppressor of cytokine signaling), two chemokine Rs, Fas, chemokines/interleukins |
|  | B11’ |  | Res 1+8 vs 0, not wt 1+8 vs 0 |  | 124 (40+, 58-) |  | Res losses: only Cyclin B1, only H1b, H4d, TLR4, TLR13  Res increases: interferon-stimulated proteins |
|  | | | | | | | |

- evidence of shut-down

** few entries, not statistically very significant.
