# Supplementary material for: Non-Apoptotic Toxicity of Pseudomonas aeruginosa toward Murine Cells
Source: PLoS One. 2013 Jan 24;8(1):e54245. doi: 10.1371/journal.pone.0054245 (PMC3554662; doi:10.1371/journal.pone.0054245)
Supplement: Table S3 — Comparison of wildtype and resistant cell responses. (DOC) [file pone.0054245.s006.doc]

**Table S3: Comparison of Wildtype and Resistant Cell Responses**

| **Protein Name** | **Res 0 *vs* Wt 0**  **from Table SB9** | **Wt 1 *vs* Wt 0**  **from Table SB1** | **Wt 1+8 *vs* Wt 0**  **from Table SB2** | **Res 1 *vs* Res 0**  **from Table SB5** | **Res 1+8 *vs* Res 0**  **from Table SB6** |  | | | | |
| --- | --- | --- | --- | --- | --- | --- | --- | --- | --- | --- |
|  |  |  |  |  |  | **Function /**  **Activity** | **Reference** | **Induced by γ-interferon ?** | | |
|  | | | | | | | | | | |
| **Relative Increase in Mutant at 1+8 h Exposure (compared to wt at 1+8 h)** | | | | | | | | | | |
| Plk2 | - | 15.8 | 15.6 | 3.2 | 65.1 | Polo-like kinase, promotes centriole division |  | |  | |
| Cxcl10 | 0.20 | 8.6 | 8.3 | 28.0 | 43.9 | Chemokine, binds Cxcr3 |  | | + | |
| Ifi202b | - | - | - | - | 39.0 | Promotes LPS/NF-kB signaling |  | | + | |
| Gbp3 | - | - | - | - | 30.7 | Guanylate binding |  | | + | |
| Ccl22 | - | - | 2.6 | 2.2 | 26.4 | Cytokine, chemoattractant, binds CCR4 |  | | + | |
| Myc | - | 5.4 | 6.3 | 5.6 | 26.3 | Promotes proliferation |  | |  | |
| Gbp6 | - | - | - | - | 19.0 | Guanylate binding |  | | + | |
| Parp14 | 0.4 | - | - | - | 12.2 | Poly(ADP-ribose) polymerase; Promotes survival |  | |  | |
| Isg20 | - | - | - | - | 10.2 | Exonuclease |  | | + | |
| Tnc | - | - | - | - | 10.0 | Tenascin, binds fibronectin, causes rounding |  | | + | |
| Gbp5 | - | - | - | - | 9.9 | Guanylate binding |  | | + | |
| Grap | - | - | - | - | 4.6 | Link between growth factor receptor and Ras |  | | - | |
| Cd14 | 0.31 | - | 2.0 | 2.2 | 4.1 | Subunit of Tlr4 |  | | - | |
| Nfkb1 | - | - | - | - | 3.3 | NF-kB |  | |  | |
| Cxcl16 | 0.20 | - | - | 2.0 | 3.0 | Cytokine |  | | + | |
| Tnip | - | - | - | - | 2.6 | TNFAIP3 interacting protein 1, inhibits NF-kB activation. |  | |  | |
| Traf5 | - | - | - | - | 2.4 | Tnf receptor-associated factor 5, activates NF-kB signaling |  | | + | |
| Prkrir | - | - | 0.45 | - | - | Repressor of inhibitor of PKR protein kinase |  | | + | |
| Tnfrsf11a | - | - | 0.44 | - | - | TNF receptor superfamily, member 11a |  | | + | |
| Nfam1 | - | 0.48 | 0.38 | - | - | Activates cytokine promoters (IL-13, TNF alpha) |  | |  | |
| Hist2h4 | - | - | 0.20 | - | - | Histone |  | |  | |
| Tmem195 | - | - | 0.20 | - | - | ? |  | |  | |
| Ranbp3l | - | - | 0.19 | - | - | ? |  | |  | |
| Prps2 | - | - | 0.19 | - | - | Purine/pyrimidine synthesis |  | |  | |
| E2f8 | - | - | 0.17 | - | - | Activates GCPRs |  | |  | |
| Slc16a12 | - | - | 0.17 | - | - | Monocarboxylic acid transport |  | |  | |
| Hist1h4f | - | - | 0.12 | - | - | Histone |  | |  | |
| Hist1h2bb | - | - | 0.12 | - | - | Histone |  | |  | |
| Stmn1 | - | - | 0.20 | - | 0.43 | Strathmin, Microtubule destabilizing |  | |  | |
| Wee1 | - | - | 0.21 | - | 0.42 | Kinase, inhibits cell cycle progression |  | | - | |
| Anln | - | - | 0.17 | - | 0.42 | Anillin, actin binding protein |  | |  | |
| Mmd | 0.49 | - | 0.14 | - | 0.34 | Monocyte to macrophage differentiation-associated |  | |  | |
| Rasgrp3 | - | - | 0.10 | - | 0.25 | RAS, guanylate exchange factor |  | |  | |
|  | | | | | | | | | | |
| **Relative Increase in Wildtype at 1+8 h Exposure (compared to Mutant at 1+8 h)** | | | | | | | | | | |
| Saa3 | - | 3.4 | 48.2 | 3.7 | 13.8 | Serum amyloid, an Acute Phase Response product |  | | | + |
| Serpin b2 | - | 2.0 | 40.2 | - | - | Plasminogen activator inhib-2 |  | | | + |
| Csf3/MIP2b | - | 2.8 | 23.6 | - | - | Cytokine |  | | | + |
| Slc7a11 | - | 6.4 | 18.7 | - | - | Transports cystine, glutamate |  | | |  |
| Edn-1 | - |  | 18.4 | - | - | Vessel constriction |  | | | + |
| Slfn4 | - | 2.5 | 14.5 | - | - | Schlafen, Induced by LPS without Myd88 |  | | | + |
| CD274/PD-L1/B7-H1 | - | - | 12.6 | - | - | Programmed cell death ligand; Immune suppression |  | | | + |
| Phlda1 | - | 10.1 | 11.6 | 4.5 | 2.8 | Anti-apoptotic ? |  | | |  |
| Gpr109a | - | 2.4 | 11.2 | - | - | GPCR for nicotine/butyrate; promotes apoptosis; opposes NF-kB activation |  | | |  |
| Serpinb9 | - | - | 10.2 | - | - | Inhibits apoptosis |  | | |  |
| Socs3 | - | 2.8 | 9.9 | - | - | Suppressor of Stat signaling, binds JAK2 |  | | | + |
| Cxcl3 | - | - | 9.0 | - | - | Cytokine |  | | | + |
| Fas | - | 3.2 | 6.5 | - | - | Pro-apoptotic |  | | | + |
| Rgs1 | 3.7 | 4.3 | 4.8 | 0.41 | 0.42 | Inhibits G-protein signaling 1; acts as a GAP, terminates the signal |  | | | + |
| Slfn10 | - | 2.3 | 3.3 | - | - | Helicase |  | | | + |
| JAK2 | - | 2.9 | 3.3 | - | - |  |  | | | + |
| Slc40a1 | 3.7 | - | 2.7 | - | - | Iron export |  | | |  |
| Nfkbie | - | 2.4 | 2.2 | - | - | Inhibits NF-kB transactivation |  | | |  |
| Cfh | - | - | - | - | 0.34 | Complement factor H |  | | |  |
| Bhlhb3 | - | - | - | 0.36 | 0.34 | Transcription factor |  | | |  |
| Kif11 | - | - | - | - | 0.34 | Kinesin 11 |  | | |  |
| Plxnd1 | 3.0 | - | - | - | 0.32 | Semaphorin receptor |  | | |  |
| S1pr1 | 2.8 | - | - | 0.44 | 0.31 | Sphingosine-phosphate receptor, Sustains STAT3 activation |  | | |  |
| Trem2 | - | - | - | - | 0.29 | Triggering receptor for activation of PI3K, ERK |  | | | - |
| Ptgds2 | 2.4 | - | - | - | 0.27 | Prostaglandin D2 synthase 2 |  | | |  |
| Tlr13 | - | - | - | - | 0.27 | Toll-like receptor 13, inhibited by NF-kB |  | | |  |
| Tlr4 | - | - | - | - | 0.36 | Toll-like receptor 4 |  | | | + |
|  | | | | | | | | | | |

| **Increased Before Exposure, Little Differential Response Upon Exposure to *Pseudomonas*** | | | | | | | | |
| --- | --- | --- | --- | --- | --- | --- | --- | --- |
| CD33 | 4.8 | - | - | - | - | Binds sialic acids |  |  |
| CD48 | 4.2 | - | - | - | - | Role in T cell activation |  | + |
| Serpinb6b | 4.1 | - | - | - | - | Peptidase inhibitor |  |  |
| Plxnc1 | 4.0 | - | - | - | - | Semaphorin receptor |  |  |
| Gpnmb | 3.6 | - | - | - | - | Osteoactivin |  |  |
| Cox-1/Ptgs-1 | 3.3 | - | 0.31 | - | - | Prostaglandin synthesis |  |  |
| CD97 | 3.3 | - | - | - | - | DAF receptor |  | - |
| Bhlhb2 | 3.2 | 6.7 | - | 2.5 | - | Transcription factor |  |  |
| CD83 | 3.2 | 10.8 | 5.5 | 5.4 | 2.6 | Antigen presentation ? |  | + |
| Plxn1 | 3.0 | - | - | - | - | Semaphorin receptor |  |  |
| Prafr-1 | 2.7 | - | - | - | - | Platelet-activating factor receptor |  |  |
| Rnase L | 2.2 | - | - | - | - | Anti-viral, activated by Oas2 |  | + |
| Tgfbr1 | 2.1 | - | - | - | - | TGFβ receptor |  | + |

|  | | | | | | | | |
| --- | --- | --- | --- | --- | --- | --- | --- | --- |
| **Reduced Before Exposure, Little Differential Response Upon Exposure to *Pseudomonas*** | | | | | | | | |
| Ifitm3 | 0.47 | - | - | - | - | Anti-viral |  | + |
| Oas2 | 0.47 | - | - | 2.2 | - | 2'-5' oligoadenylate synthetase 2, activates RNase L |  | + |
| Tlr8 | 0.45 | - | - | - | - | Toll-like receptor, responds to oligonucleotides |  |  |
| Ifih1 | 0.42 | 2.5 | 6.7 | 2.7 | 12.2 | Anti-viral helicase |  | + |
| Il6st | 0.42 | - | - | - | - | Cytokine receptor, functions with JAK/STAT |  | + |
| Irf2 | 0.41 | - | - | - | - | Inhibits interferon type I induction |  | + |
| Ifi44 | 0.40 | 0.50 | - | - | - | Microtubule associated ? |  | + |
| Ptger-4 | 0.39 | - | - | - | - | Prostaglandin E4 receptor |  |  |
| Ifi27 | 0.35 | 0.44 | - | - | - | Promotes apoptosis |  | + |
| Irf7 | 0.29 | - | - | - | - | Activates interferon type I induction |  |  |
| Cox-2/Ptgs-2 | 0.25 | 5.4 | 12.0 | 5.8 | 36.6 | Prostaglandin-endoperoxide synthase 2 |  | + |
| CD44 | 0.11 | - | - | - | - | Binds hyaluronic acid, osteopontin, collagens, MMPs |  | + |
|  | | | | | | | | |
| **Various** | | | | | | | | |
| CD80/B7-1 | 7.2 | - | 5.2 | 2.0 | 3.3 | Binds PD-L1 (programmed cell death ligand-1) |  | + |
| Ifrd1 | - | 3.4 | 2.0 | 2.9 | - | Binds NF-kB, HDAC3/4; promotes muscle differentiation |  |  |
| Il1rn | - | - | 22.1 | 4.2 | 9.1 |  |  |  |
| Irf4 | - | 2.8 | - | - | 2.1 | Transcription factor, binds STAT6, negatively regulates NF-kB signaling |  |  |
| Nfkb2 | - | 2.8 | 2.7 | - | 3.2 | NF-kB p100 subunit |  |  |
| Nr4a1 | - | - | - | 12.1 | - | Nuclear receptor subfamily 4, group A, member 1 |  |  |
| Tnfrsf1b | - | 2.8 | 16.5 | - | 13.9 | TNF receptor superfamily, member 1b |  |  |
| Tnfsf9 | - | 4.0 | - | 4.3 | 3.0 | TNF superfamily, member 9 |  |  |

**References**

1. Shi Z, Cai Z, Wen S, Chen C, Gendron C, et al. (2009) Transcriptional regulation of the novel Toll-like receptor Tlr13. J Biol Chem 284: 20540-20547.

2. Dufour JH, Dziejman M, Liu MT, Leung JH, Lane TE, et al. (2002) IFN-gamma-inducible protein 10 (IP-10; CXCL10)-deficient mice reveal a role for IP-10 in effector T cell generation and trafficking. J Immunol 168: 3195-3204.

3. Choubey D, Kotzin BL (2002) Interferon-inducible p202 in the susceptibility to systemic lupus. Front Biosci 7: e252-262.

4. Ludlow LE, Purton LE, Klarmann K, Gough DJ, Hii LL, et al. (2008) The role of p202 in regulating hematopoietic cell proliferation and differentiation. J Interferon Cytokine Res 28: 5-11.

5. Vestal DJ, Jeyaratnam JA (2011) The guanylate-binding proteins: emerging insights into the biochemical properties and functions of this family of large interferon-induced guanosine triphosphatase. J Interferon Cytokine Res 31: 89-97.

6. Ghadially H, Ross XL, Kerst C, Dong J, Reske-Kunz AB, et al. (2005) Differential regulation of CCL22 gene expression in murine dendritic cells and B cells. J Immunol 174: 5620-5629.

7. Aguiar RC, Takeyama K, He C, Kreinbrink K, Shipp MA (2005) B-aggressive lymphoma family proteins have unique domains that modulate transcription and exhibit poly(ADP-ribose) polymerase activity. J Biol Chem 280: 33756-33765.

8. Cuzzocrea S (2005) Shock, inflammation and PARP. Pharmacol Res 52: 72-82.

9. Degols G, Eldin P, Mechti N (2007) ISG20, an actor of the innate immune response. Biochimie 89: 831-835.

10. Udalova IA, Ruhmann M, Thomson SJ, Midwood KS (2011) Expression and immune function of tenascin-C. Crit Rev Immunol 31: 115-145.

11. Shen R, Ouyang YB, Qu CK, Alonso A, Sperzel L, et al. (2002) Grap negatively regulates T-cell receptor-elicited lymphocyte proliferation and interleukin-2 induction. Mol Cell Biol 22: 3230-3236.

12. Takamatsu H, Okuno T, Kumanogoh A (2010) Regulation of immune cell responses by semaphorins and their receptors. Cell Mol Immunol 7: 83-88.

13. Kawasaki M, Fujishiro M, Yamaguchi A, Nozawa K, Kaneko H, et al. (2010) Fluctuations in the gene expression of peripheral blood mononuclear cells between the active and inactive phases of systemic lupus erythematosus. Clin Exp Rheumatol 28: 311-317.

14. Nakano H, Oshima H, Chung W, Williams-Abbott L, Ware CF, et al. (1996) TRAF5, an activator of NF-kappaB and putative signal transducer for the lymphotoxin-beta receptor. J Biol Chem 271: 14661-14664.

15. Gale M, Jr., Blakely CM, Hopkins DA, Melville MW, Wambach M, et al. (1998) Regulation of interferon-induced protein kinase PKR: modulation of P58IPK inhibitory function by a novel protein, P52rIPK. Mol Cell Biol 18: 859-871.

16. Ohtsuka M, Arase H, Takeuchi A, Yamasaki S, Shiina R, et al. (2004) NFAM1, an immunoreceptor tyrosine-based activation motif-bearing molecule that regulates B cell development and signaling. Proc Natl Acad Sci U S A 101: 8126-8131.

17. Hagemann IS, Narzinski KD, Baranski TJ (2007) E2F8 is a nonreceptor activator of heterotrimeric G proteins. J Mol Signal 2: 3.

18. Ringhoff DN, Cassimeris L (2009) Stathmin regulates centrosomal nucleation of microtubules and tubulin dimer/polymer partitioning. Mol Biol Cell 20: 3451-3458.

19. Neumann B, Zhao L, Murphy K, Gonda TJ (2008) Subcellular localization of the Schlafen protein family. Biochem Biophys Res Commun 370: 62-66.

20. van Zuylen WJ, Garceau V, Idris A, Schroder K, Irvine KM, et al. (2011) Macrophage activation and differentiation signals regulate schlafen-4 gene expression: evidence for Schlafen-4 as a modulator of myelopoiesis. PLoS One 6: e15723.

21. Johnson EO, Chang KH, de Pablo Y, Ghosh S, Mehta R, et al. (2011) PHLDA1 is a crucial negative regulator and effector of Aurora A kinase in breast cancer. J Cell Sci 124: 2711-2722.

22. Li G, Shi Y, Huang H, Zhang Y, Wu K, et al. (2010) Internalization of the human nicotinic acid receptor GPR109A is regulated by G(i), GRK2, and arrestin3. J Biol Chem 285: 22605-22618.

23. Kummer JA, Micheau O, Schneider P, Bovenschen N, Broekhuizen R, et al. (2007) Ectopic expression of the serine protease inhibitor PI9 modulates death receptor-mediated apoptosis. Cell Death Differ 14: 1486-1496.

24. Tamiya T, Kashiwagi I, Takahashi R, Yasukawa H, Yoshimura A (2011) Suppressors of cytokine signaling (SOCS) proteins and JAK/STAT pathways: regulation of T-cell inflammation by SOCS1 and SOCS3. Arterioscler Thromb Vasc Biol 31: 980-985.

25. Rainard P, Riollet C, Berthon P, Cunha P, Fromageau A, et al. (2008) The chemokine CXCL3 is responsible for the constitutive chemotactic activity of bovine milk for neutrophils. Mol Immunol 45: 4020-4027.

26. Moratz C, Kang VH, Druey KM, Shi CS, Scheschonka A, et al. (2000) Regulator of G protein signaling 1 (RGS1) markedly impairs Gi alpha signaling responses of B lymphocytes. J Immunol 164: 1829-1838.

27. Tran T, Paz P, Velichko S, Cifrese J, Belur P, et al. (2010) Interferonbeta-1b Induces the Expression of RGS1 a Negative Regulator of G-Protein Signaling. Int J Cell Biol 2010: 529376.

28. Mayr R, Griffiths WJ, Hermann M, McFarlane I, Halsall DJ, et al. (2011) Identification of mutations in SLC40A1 that affect ferroportin function and phenotype of human ferroportin iron overload. Gastroenterology 140: 2056-2063, 2063 e2051.

29. Li Z, Nabel GJ (1997) A new member of the I kappaB protein family, I kappaB epsilon, inhibits RelA (p65)-mediated NF-kappaB transcription. Mol Cell Biol 17: 6184-6190.

30. Yamada K, Miyamoto K (2005) Basic helix-loop-helix transcription factors, BHLHB2 and BHLHB3; their gene expressions are regulated by multiple extracellular stimuli. Front Biosci 10: 3151-3171.

31. Lee H, Deng J, Kujawski M, Yang C, Liu Y, et al. (2010) STAT3-induced S1PR1 expression is crucial for persistent STAT3 activation in tumors. Nat Med 16: 1421-1428.

32. Kono M, Mi Y, Liu Y, Sasaki T, Allende ML, et al. (2004) The sphingosine-1-phosphate receptors S1P1, S1P2, and S1P3 function coordinately during embryonic angiogenesis. J Biol Chem 279: 29367-29373.

33. Peng Q, Malhotra S, Torchia JA, Kerr WG, Coggeshall KM, et al. (2010) TREM2- and DAP12-dependent activation of PI3K requires DAP10 and is inhibited by SHIP1. Sci Signal 3: ra38.

34. Turnbull IR, Gilfillan S, Cella M, Aoshi T, Miller M, et al. (2006) Cutting edge: TREM-2 attenuates macrophage activation. J Immunol 177: 3520-3524.

35. Micheli L, Leonardi L, Conti F, Maresca G, Colazingari S, et al. (2011) PC4/Tis7/IFRD1 stimulates skeletal muscle regeneration and is involved in myoblast differentiation as a regulator of MyoD and NF-kappaB. J Biol Chem 286: 5691-5707.

36. Paun A, Pitha PM (2007) The innate antiviral response: new insights into a continuing story. Adv Virus Res 69: 1-66.

37. Bonfield TL, Thomassen MJ, Farver CF, Abraham S, Koloze MT, et al. (2008) Peroxisome proliferator-activated receptor-gamma regulates the expression of alveolar macrophage macrophage colony-stimulating factor. J Immunol 181: 235-242.
