# Supplementary material for: Non-Apoptotic Toxicity of Pseudomonas aeruginosa toward Murine Cells
Source: PLoS One. 2013 Jan 24;8(1):e54245. doi: 10.1371/journal.pone.0054245 (PMC3554662; doi:10.1371/journal.pone.0054245)
Supplement: Table S4 — Summary of Relative Changes After 1+8 h Exposure to Pseudomonas aeruginosa . (DOC) [file pone.0054245.s007.doc]

**Table S4: Summary of Relative Changes After 1+8 h Exposure to *Pseudomonas***

| Relative Increases in the Mutant | Relative Decreases in the Mutant |
| --- | --- |
| **Expected Impact on TLR Signaling in the Mutant** | |
| Positive: CD14, If202b, Nfkb1, Traf5 | Nfkbie, Gpr109a |
| Negative: Tnip | Tlr4, Tlr13 |
|  | |
| **Expected Impact on JAK/STAT Signaling in the Mutant** | |
| Positive: Grap, Nfam1 | Socs3 |
| Negative: | S1pr1 |
|  | |
| **Expected Impact on GPCR Signaling in the Mutant** | |
| Positive: E2f8 | Rgs1, Trem2 |
|  | |
| **Expected Impact on Growth of the Mutant** | |
| Positive: Gbp3, Gbp5, Gbp6, Myc, Parp14, Plk2, Pkrir, Rasgrp3 | Cd274, Fas, Phda1, Serpin b9 |
| Negative: Wee1 |  |
|  | |
| **Impact on Rounding ?** | |
| Tnc |  |
|  | |
| **Protaglandin-Related ?** | |
|  | Ptgds2 |
|  | |
| **Cytokines** | |
| Cxcl10, Cxcl16, Cxcl22 | Cxcl3, Il1f6 |
|  | |
| **Other – Significance Unknown** | |
| Anln, Isg20, Mmd, Nfam1, Pkrir, Prps2, Ranbp31, Slc16a12, Stmn1, Tmem195, TNFrsf11a, Tnfrsf12a | Bhlhb3, Caf3, Cfh, Edn-1, Kif1, Plxnd1, Saa3, Serpinb2, Slc7a11, Slc40a, Slfn4, Slfn10, Stmn1, Tlr13 |
